# Orychophragvioline A, a Novel Alkaloid Isolated from *Orychophragmus violaceus* with Anti-Cervical Cancer Activity

**DOI:** 10.3390/molecules30081759

**Published:** 2025-04-14

**Authors:** Ya Li, Tonghe Liu, Guangjie Pan, Yihang Li, Guoxu Ma, Yong Hou, Nailiang Zhu, Xudong Xu

**Affiliations:** 1 Department of Gynecologic Cancer, Beijing Arion Cancer Center, Beijing 100070, China; yaya5012@163.com; 2 Key Laboratory of Bioactive Substances and Resource Utilization of Chinese Herbal Medicine, Ministry of Education, Institute of Medicinal Plant Development, Peking Union Medical College and Chinese Academy of Medical Sciences, Beijing 100193, China; 3 College of Chinese Medicine Material, Jilin Agricultural University, Changchun 130118, China; 4 Yunnan Key Laboratory of Southern Medicine Utilization, Yunnan Branch, Institute of Medicinal Plant Development, Peking Union Medical College and Chinese Academy of Medical Sciences, Jinghong 666100, China; 5 Department of Traditional Chinese Medicine Resources and Development, School of Pharmacy, Xinyang Agricultural and Forestry University, Xinyang 464000, China

**Keywords:** orychophragvioline A, natural product, anti-cervical cancer, new compound

## Abstract

A new alkaloid (orychophragvioline A) and nine known compounds were yielded from the seeds of *Orychophragmus violaceus*. Their structures were determined by various spectroscopic techniques and single-crystal X-ray diffraction. Orychophragvioline A is a rare alkaloid with an unusual 1-methyl-4-phenyl-2,3-diketopiperazine skeleton connected with a guanidine group via an amide bond. The results of antitumor tests in vitro showed that it exhibited prominent cytotoxicity against Hela cells with an IC_50_ value of 11.95 ± 1.52 μM. Further investigations suggested that it significantly inhibited cellular proliferation, migration, and invasion in a dose-dependent manner, thus inducing the apoptosis of Hela cells. These findings indicate that orychophragvioline A can be regarded as a potential natural leading compound against cervical cancer.

## 1. Introduction

Natural products, especially those derived from plants, have long been recognized as a primary source of new drugs and as lead compounds for treating various diseases [1]. In terms of new antitumor drugs approved by the US Food and Drug Administration (FDA) from 1981 to 2019, relevant natural products accounted for nearly 33.5% [2]. Some natural products, such as paclitaxel, homoharringtonine, and camptothecin, have been applied in clinics for cancer therapy [3,4,5]. Thus, the discovery of novel natural products from various plants is of great significance in the field of the research and development of anti-cancer drugs. 

*Orychophragmus violaceus* (L.) is a common medicinal plant belonging to the Brassicaceae family, which is also used as a wild vegetable or ornamental plant. It is widely distributed in most areas of north China, like plains, mountain slopes, and roadsides. The whole herb contains rich nutrients including vitamins, protein, and microelements. The seeds have the characteristics of a high oil content, offering a highly valuable oil crop. Historically, this plant has been seen as a traditional folk medicine. Modern pharmacological studies show that it exhibits multiple pharmacological effects, embracing antioxidant, anti-inflammatory, antitumor, and liver-protecting properties [6]. There are excellent efficiency benefits from the diverse active ingredients existing in *O. violaceus*. Previous investigations displayed that over one hundred chemical constituents have been isolated from this plant, comprising alkaloids, flavonoids, saponin, isocoumarins, lignans, and terpenoids [7,8,9,10,11,12]. Among them, three novel compounds bearing a 2-piperazinone-fused 2,4-dioxohexahydro-1,3,5-triazine skeleton have drawn great attention, which might become promising leading drug candidates against cancers [8]. Hence, it is worth exploring more similar new compounds with potential cancer-fighting properties from *O. violaceus*.

**Figure 1:**
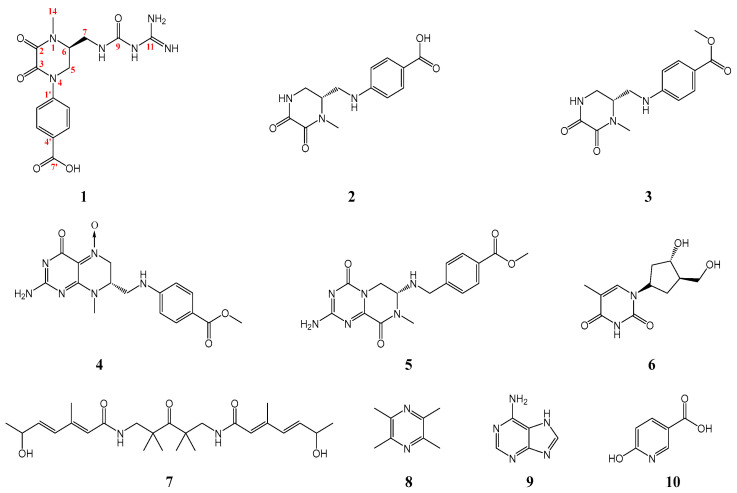
Chemical structures for compounds **1**–**10**.

In this study, one new and nine known alkaloid compounds (Figure 1) were yielded from the seeds of *O. violaceus*, which were identified as orychophragvioline A (**1**), demethylorychophragine A (**2**), orychophragine A (**3**), pilealkaloid A (**4**), orychophragine D (**5**), thymidine (**6**), arboreumine (**7**), tetramethylpyrazine (**8**), adenine (**9**), and 6-hydroxynicotinic acid (**10**). Furthermore, the novel compounds had been found to exhibit excellent antiproliferative activity against Hela cells, which might make them an efficacious natural alternative candidate in the treatment of patients with cervical cancer.

## 2. Results and Discussion

### 2.1. Structural Elucidation of Orychophragvioline A

Orychophragvioline A (**1**) was obtained as colorless tabular crystals, which were easily soluble in dimethyl sulfoxide (DMSO), but insoluble in water or methanol. The IR spectrum showed characteristic peaks assignable to N–H (3376 cm^−1^), O-H (3513 cm^−1^), C=O (1656 cm^−1^), and aromatic ring (3016 cm^−1^, 1425 cm^−1^, and 788 cm^−1^) functional groups. The HR-ESI–MS gave a molecular formula of C_15_H_18_N_6_O_5_ at *m*/*z* 363.1415 [M + H]^+^ (calcd for C_15_H_19_N_6_O_5_, 363.1411), with 9 degrees of unsaturation.

**Figure 2:**
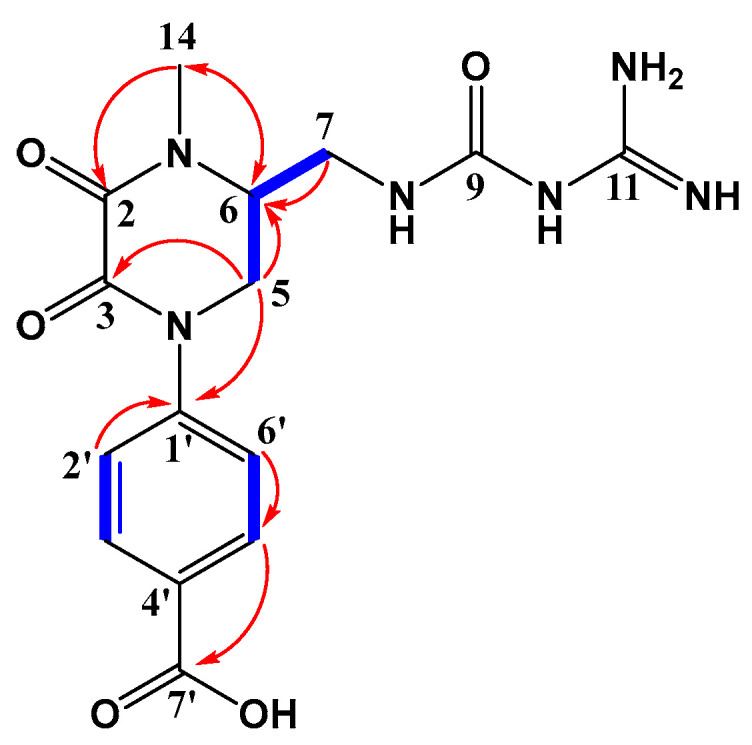
Key ^1^H-^1^H COSY (bold lines) and HMBC (arrows) correlations of compound **1**.

The ^1^H-NMR spectrum (DMSO-*d_6_*, 600 MHz) showed a methyl proton signal at *δ_H_* 3.07 (3H, s, 14-CH_3_), which suggested the presence of an *N*-methyl. The typical proton signals for the AA′BB′-type aromatic ring at *δ_H_* 7.48 (2H, d, *J* = 8.3 Hz, H-2′, 6′) and 7.89 (2H, d, *J* = 8.3 Hz, H-3′, 5′) showed that there were 1,4-disubstituted phenyl rings in compound **1**. Moreover, ^1^H-NMR data showed characteristic proton signals of one methine at *δ_H_* 3.39 (1H, m, H-5b) and two methylene at *δ_H_* 3.67 (3H, m, H-5a, 6, 7b) and *δ_H_* 4.33 (1H, dd, *J* = 12.9, 3.7 Hz, H-7a). Meanwhile, the ^13^C-NMR spectrum (DMSO-*d_6_*, 150 MHz) also presented the corresponding carbon signals of the 1,4-disubstituted phenyl ring at *δ_C_* 143.0 (C-1′), 124.8 (C-2′, 6′), 129.9 (C-3′, 5′), and 135.4 (C-4′), and an *N*-methyl group at *δ_C_* 33.5 (C-14). Additionally, four carbonyl carbon signals at *δ_C_* 156.5 (C-2, 11) and 155.9 (C-3, 9), a methine carbon signal at *δ_C_* 54.8 (C-6), and two methylene carbon signals at *δ_C_* 39.3 (C-5) and 48.1 (C-7) were revealed in the ^13^C-NMR spectrum. From the ^1^H–^1^H COSY and HMBC spectrum (Figure 2), pairwise correlations among H-5, H-6, and H-7, combined with the correlations from H_3_-14 to C-2/C-6, from H-5 to C-3/C-6, and from H-7 to C-6, indicated a 1-methyl-2,3-diketopiperazine fragment in compound **1**, and the correlations from H-5 to C-1′ and from H-3′ to C-7′ inferred that the 2,3-diketopiperazine fragment was connected to the phenyl ring *p*-substituted by a carboxyl group. The total assignment of the hydrocarbon signals of compound **1** were displayed in Table 1 by combining the aforementioned spectral data with the HSQC spectrum (Supplementary Materials Figures S1–S8).

**Figure 3:**
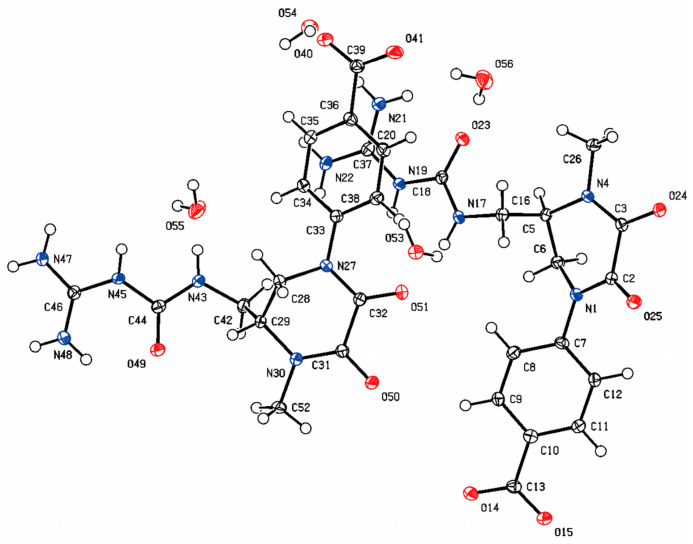
ORTEP drawing of the crystal structure of orychophragvioline A.

Considering the number of N atoms could not be clearly defined by the present data, compound **1** was recrystallized from different solvents in order to confirm the structure and absolute configuration. Fortunately, the crystal of compound **1** was observed from a mixed solvent of methanol/water/formic acid. On the basis of a Flack parameter value of −0.05 (5), single-crystal X-ray diffraction analysis (CCDC 2422978) verified its absolute configuration as 6*R* (Figure 3 and Supplementary Materials Table S1). Thus, compound **1** was definitely established and named as orychophragvioline A.

From Figure 3, we find that compound **1** exists as a dimer, which may be related to the guanidyl and carboxyl groups. The guanidyl group were protonated and had a positive charge, while the carboxyl group was the opposite. Thus, the strong intermolecular electrostatic interaction was induced by the opposite charges, which also prompted the formation of crystal. The alkaloid structure with a 1-methyl-4-phenyl-2,3-diketopiperazine skeleton connected with a guanidine group via an amide bond and the dimer crystal form are both rare in natural small molecules. In addition, nine known alkaloid compounds (2–10, Figure 1 and Supplementary Materials Figures S9–S26)) were isolated and identified by comparing them with the literature, including demethylorychophragine A (**2**) [13], orychophragine A (**3**) [8], pilealkaloid A (**4**) [14], orychophragine D (**5**) [15], thymidine (**6**) [16], arboreumine (**7**) [17], tetramethylpyrazine (**8**) [18], adenine (**9**) [19], and 6-hydroxynicotinic acid (**10**) [20]. Various chromatographic techniques such as macroporous resin column chromatography, Sephadex LH-20, reversed-phase chromatography, and semi-preparative HPLC assistant with traditional methods like recrystallization were used for the separation and purification of these compounds. Comparatively, the abundance of common alkaloids like thymidine and adenine was the highest and the next highest was piperazine alkaloids. As one of the main components of the seeds of *O. violaceus*, the rich variety and content of alkaloids could produce more diversified new active substances.

### 2.2. Proposed Biosynthetic Pathway for Orychophragvioline A

**Scheme 1:**
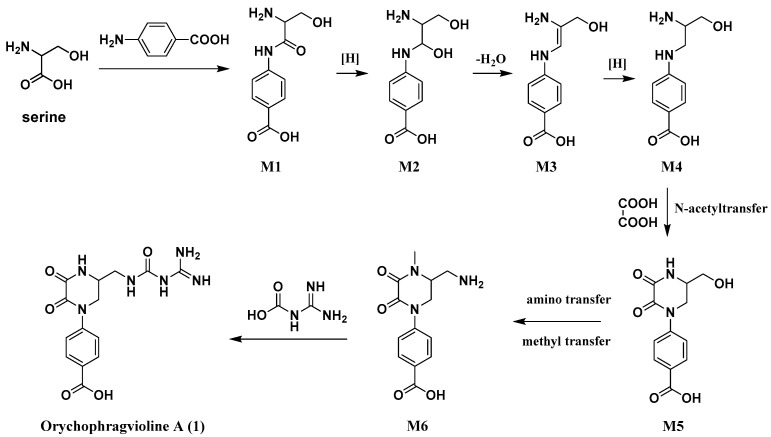
Proposed biosynthetic pathway for orychophragvioline A (**1**).

The rare skeletal structure of orychophragvioline A engaged our interest in its biosynthetic pathway. A plausible biogenetic pathway for it is shown in Scheme 1. Serine and *p*-aminobenzoic acid were considered the biogenetic precursors, which produced amide bonds through dehydration condensation to form intermediate **M1**. Following a series of reductions and dehydration, intermediate **M1** would transform into **M4**. Then, intermediate **M4** reacted with oxalic acid to generate **M5** by the enzymic catalytic reaction of *N*-acyltransferase. Using the aminotransferase and methyltransferase, intermediate **M6** was derived from intermediate **M5**. Finally, orychophragvioline A was obtained from the reaction between **M6** and guanidine formic acid.

### 2.3. Cytotoxicity Evaluation of Orychophragvioline A

Inspired by previously reported natural alkaloids exhibiting remarkable cytotoxicity, the cytotoxic activity in vitro of orychophragvioline A against several cell lines, including the Hela (human cervical cancer cell), U251 (human glioma cell), SW1990 (human pancreatic cancer cell), 4T1 (mouse breast cancer cell), and L02 (human normal liver cell) cell lines, were evaluated by the CCK8 method. Cisplatin was used as the positive control. As shown in Table 2, orychophragvioline A exhibited obvious cytotoxicity against the cancer cell lines, with the strongest anti-cancer activity seen in Hela (IC_50_ values of 11.95 ± 1.52 μM). Therefore, the Hela cell line was chosen to further assess the mechanism of action of orychophragvioline A.

### 2.4. Orychophragvioline A-Induced Apoptosis of Hela Cells

**Figure 4:**
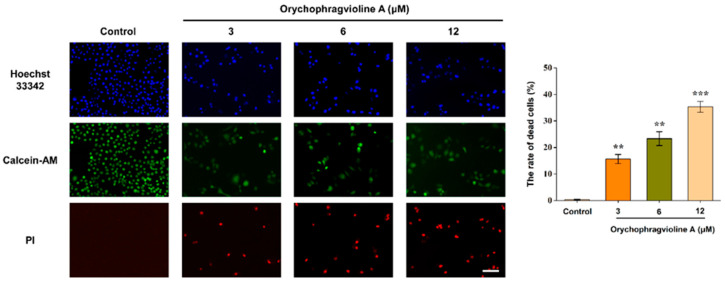
The live/dead fluorescent staining images and the corresponding semi-quantitative measurement of Hela cells treated with orychophragvioline A (0, 3, 6, and 12 µM) for 48 h. Scale bar: 100 µm. ** *p* < 0.01, *** *p* < 0.001 vs. control group.

Apoptosis, also termed as programmed cell death, is regarded as a paramount process for eliminating cancer cells. The vast majority of chemotherapy drugs kills cancer cells through promoting apoptosis [21]. To further evaluate the apoptosis and necrosis of Hela cells caused by orychophragvioline A, we performed a live/dead staining experiment. As shown in Figure 4, we observed that it could lead to cell death along with the loss of nuclei in a dose-dependent manner, according to the calcein-AM, ropidium iodide (PI), and Hoechst 33342 staining. As a fluorescent dye that binds to DNA, staining with Hoechst 33342 will present the nuclear changes in apoptosis. Compared to the control group, we found that orychophragvioline A could cause nuclear fragmentation, particularly at a higher concentration. According to statistical results, the cell death rates in the treatment groups were all significantly higher than that of the control group, which was also in good agreement with the aforementioned CCK-8 results.

**Figure 5:**
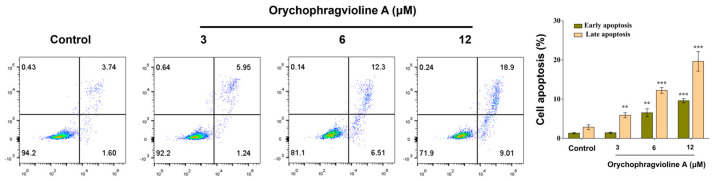
Flow cytometric analysis of apoptosis rates and the corresponding semiquantitative measurement of Hela cells treated with orychophragvioline A (0, 3, 6, and 12 µM) for 48 h with Annexin V-FITC/PI staining. ** *p* < 0.01, *** *p* < 0.001 vs. control group.

Next, flow cytometric analysis was applied for quantitatively examining the different stages of apoptosis of Hela cells treated by orychophragvioline A at different doses. Except for the low-dose group, the percentage of apoptosis in both the early and late phases increased markedly in all groups. Especially in the late phase, the rate of apoptosis in the high-dose group could reach 18.9% (Figure 5). These findings collectively evidenced that orychophragvioline A could strikingly induce apoptosis in the Hela cells.

### 2.5. Orychophragvioline A Inhibits the Colony Formation of Hela Cells

**Figure 6:**
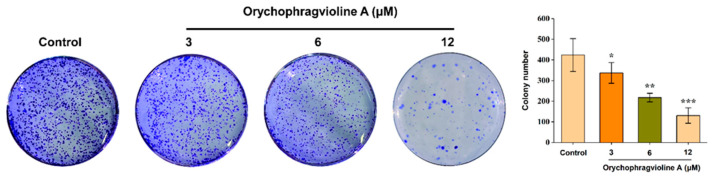
The proliferation ability and semi-quantitation in bar graphs of Hela cells treated with orychophragvioline A (0, 3, 6, and 12 µM) for 10 d by colony formation assay (*n* = 3). Data are shown as the mean ± SD. * *p* < 0.05, ** *p* < 0.01, *** *p* < 0.001 vs. control group.

The cell colony formation assay is usually used to evaluate the long-term toxicity of compounds through observing the cell colony number. We assessed the antiproliferative effect of orychophragvioline A utilizing this method. As displayed in Figure 6, we found that the colony number of Hela cells apparently decreased with the increase in concentration. In the control group, the cell colony number was about 424. After treatment with orychophragvioline A, it was reduced significantly and even shrank by approximately 60% in the high-dose group. The findings supported that orychophragvioline A had a strong ability to inhibit the proliferation of Hela cells over an extended duration.

### 2.6. Orychophragvioline A Suppresses the Migration and Invasion of Hela Cells

**Figure 7:**
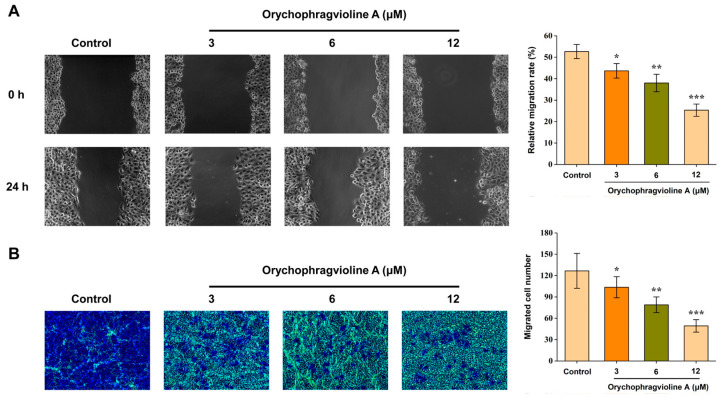
The migration ability and semi-quantitation in bar graphs of Hela cells treated with orychophragvioline A (0, 3, 6, and 12 µM) for 24 h by wound healing (**A**) and transwell migration (**B**) assay (*n* = 3). Data are shown as the mean ± SD. * *p* < 0.05, ** *p* < 0.01, *** *p* < 0.001 vs. control group.

The wound healing and transwell migration assay aimed to simulate cancer cell migration and invasion in vivo. Thus, we further evaluated the ability of orychophragvioline A to inhibit the migration and invasion of Hela cells. After 24 h of treatment, the scratch width of the Hela cells in the control group had reduced significantly (Figure 7). In contrast, orychophragvioline A could inhibit the scratch healing strikingly in a dose-dependent manner. Compared to the control group, the relative migration rate after treatment with orychophragvioline A decreased notably. When the concentration of orychophragvioline A was 12 μM, the relative migration rate decreased to about 25%. Meanwhile, a similar result was obtained from the transwell assay, where the number of migrating Hela cells was inversely proportional to the concentration of orychophragvioline A. These results indicated that orychophragvioline A could suppress the migration and invasion of Hela cells.

## 3. Materials and Methods

### 3.1. General Experimental Procedures

Optical rotation data were recorded on a Perkin-Elmer 341 digital polarimeter (PerkinElmer, Waltham, MA, USA). The UV spectra of the sample solutions were recorded on a UV-vis spectrophotometer (UV-3600, Shimadzu, Kyoto, Japan) with a range of 200–400 nm. FT-IR spectra of the samples were recorded by an FT-IR spectrometer (Nicolet IS10, ThermoFisher, Waltham, MA, USA) with a range from 4000 to 400 cm^−1^. The 1D- and 2D-NMR spectra were conducted on a Bruker AV III 600 NMR spectrometer (Bruker, Karlsruhe, Germany) with TMS as an internal standard (JMOD: C and CH_2_ up, CH_3_ and CH down). HR-ESI-MS data were performed on an LTQ-Orbitrap XL spectrometer (Thermo, Waltham, MA, USA). The X-ray crystallographic data of samples were recorded by XtaLAB Synergy (Rigaku Corporation, Akishima-shi, Japan) and X-ray single crystal diffractometers (Agilent Xcalibur Eos Gemini E, Santa Clara, CA, USA) using graphite-monochromatic Cu Kα radiation. CC (column chromatography) was carried out by silica gel (200–300 mesh, Qingdao Marine Chemical Plant, Qingdao, China) and polymer reversed-phase chromatography (MCI GEL™, Mitsubishi Chemical Corporation, Tokyo, Japan). Semi-preparative HPLC was conducted using an HPLC PUMP K-501 and LC3000 high-performance liquid chromatograph (Beijing Innovation Tong Heng Technology Co., Ltd., Beijing, China) with a Kromasil 100-5C_18_ (250 × 10 mm, E108850) column. Precoated silica gel GF254 plates (Qingdao Marine Chemical Factory, Qingdao, China) were used for thin-layer chromatography (TLC). All used solvents (Beijing Chemical Works, Beijing, China) were of an analytical grade.

### 3.2. Plant Material

The seeds of *O. violaceus* were collected in Beijing (40° N and 116° E), China, and identified by Prof. Jun Chen (Institute of Medicinal Plant Development, Peking Union Medical College and Chinese Academy of Medical Sciences). A voucher specimen has been deposited at the Laboratory of Institute of Medicinal Plant Development.

### 3.3. Extraction and Isolation

**Scheme 2:**
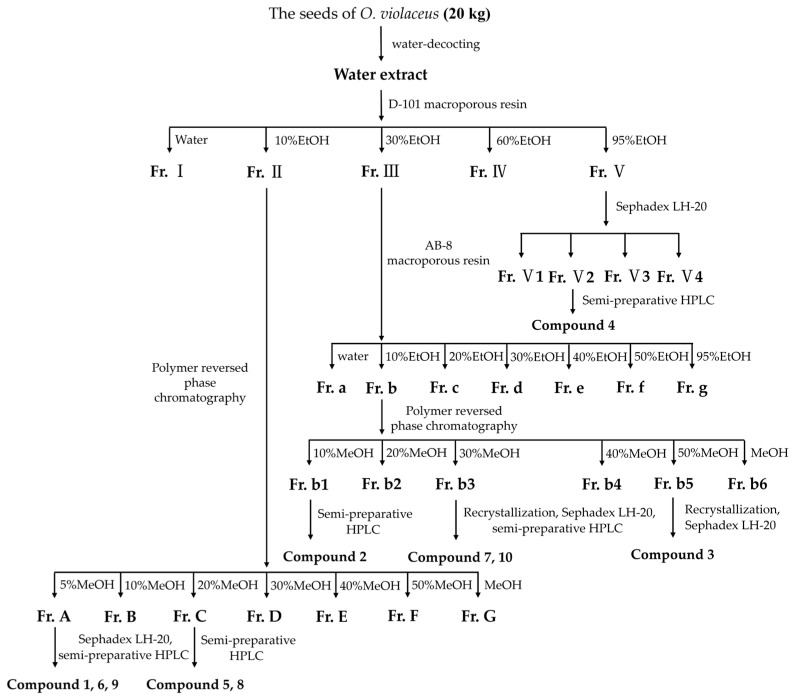
Separation and purification steps for the seeds of *O. violaceus*.

The seeds of *O. violaceus* (20 kg) were decocted with a ten-fold amount of water two times, for 1 h each time (Scheme 2). After the evaporation of the solvent, the residue (2 kg, 10% DM) was suspended in water and subjected to D-101 macroporous resin column chromatography eluted with EtOH–water gradient (0%, 10%, 30%, 60%, and 95%) to obtain five fractions (I-V). Fr. II (100 g) was subjected to polymer reversed-phase chromatography eluted with MeOH–water gradient (5%, 10%, 20%, 30%, 40%, 50%, and 100%) to yield seven fractions (Fr. A-G). Fr. A (15 g) was subjected to Sephadex LH-20 (50% MeOH) and separated with semi-preparative HPLC (30% MeOH-H_2_O with isocratic elution) to afford compound **1** (6.4 mg, 0.32 mg/kg DM), **6** (35.2 mg, 1.76 mg/kg DM), and **9** (28.6 mg, 1.43 mg/kg DM). Fr. C (11 g) was separated by semi-preparative HPLC (40% MeOH-H_2_O with isocratic elution) to yield compound **5** (5.2 mg, 0.26 mg/kg DM) and **8** (4.2 mg, 0.21 mg/kg DM). Fr. III (80 g) was subjected to AB-8 macroporous resin column chromatography eluted with EtOH–water gradient (0%, 10%, 20%, 30%, 40%, 50%, and 95%) to yield seven fractions (Fr. a-g). Fr. b (9 g) was subjected to polymer reversed-phase chromatography eluted with MeOH–water gradient (10%, 20%, 30%, 40%, 50%, and 100%) to yield six fractions (Fr. b1–6). Fr. b1 (0.8 g) was separated by semi-preparative HPLC (35% MeOH-H_2_O with isocratic elution) to yield compound **2** (15.6 mg, 0.78 mg/kg DM). Compound **7** (3.1 mg, 0.155 mg/kg DM) and **10** (3.0 mg, 0.15 mg/kg DM) were obtained by recrystallization(methanol) with Sephadex LH-20 gel chromatography and semi-preparative HPLC (45% MeOH-H_2_O with isocratic elution) from Fr. b3 (0.6 g). Fr. b5 (0.5 g) was subjected to Sephadex LH-20 gel chromatography repeatedly and recrystallization(methanol) to yield compound **3** (10.8 mg, 0.54 mg/kg DM). Fr. V (60 g) was subjected to Sephadex LH-20 gel chromatography to give four fractions (Fr. V1–4). Fr. V2 (0.1 g) was separated by semi-preparative HPLC (40% MeOH-H_2_O with isocratic elution) to yield compound **4** (25.8 mg, 1.29 mg/kg DM).

#### Orychophragvioline A (**1**)

Colorless, flaky crystal; [α]$\frac{25}{D}$ = +21.4 (c 0.1, MeOH); UV (MeOH) λmax (log ε) 295 (0.195) nm; IR (KBr) ν_max_ 3546, 3513, 3376, 3333, 3016, 2782, 1656, 1425, and 788 cm^−1^; ^1^H and ^13^C NMR data, see Table 1; HR-ESI-MS *m*/*z* 363.1415 [M + H]^+^ (calcd for C_15_H_18_N_6_O_5_, 363.1411).

### 3.4. Cell Culture and Cytotoxicity Evaluation

Hela, U251, SW1990, 4T1, and L02 cells were obtained from the Institute of Medicinal Plant Development, Peking Union Medical College and Chinese Academy of Medical Sciences (Beijing, China). All of the cell lines were cultured in Dulbecco’s modified Eagle medium (DMEM) with 10% fetal bovine serum (FBS) and 100 U/mL penicillin–streptomycin. The cells were maintained in a humidified chamber with 5% CO_2_ at 37 °C. The cells were seeded into 96-well plates at a density of 3 × 10^3^ cells/well and treated with different concentrations of test compounds (50 µM, 25 µM, 12.5 µM, 6.25 µM, and 3.13 µM). After 12 h of incubation, the cells were treated with the tested samples. Cell counting kit-8 (CCK-8, TargetMol, Boston, USA) was employed to assess cell viability. Furthermore, after being cultured in 24-well plates and treated with compound 1 for 48 h, the Hela cells were stained with Calcein-AM, PI (Beyotime, Shanghai, China), and Hoechst 33342 (Solarbio, Beijing, China). 

### 3.5. Flow Cytometry Analysis

After being cultured for 12 h in 6-well plates at a density of 5 × 10^4^ cells/well, compound 1 was added to coculture with Hela cells for 48 h. Subsequently, the cells were harvested and washed with PBS. Then, the Annexin V-PI staining apoptosis kit was used to analyze the apoptosis rate of the cells according to the instructions.

### 3.6. Colony Formation Assay

Hela cells were seeded in a 6-well plate (1 × 10^3^ cells/well), treated with the tested samples, and cultured for 10 days in a 35% CO_2_ environment. Colonies of Hela cells were fixed, stained, and counted (10^5^ cells per colony) under a microscope using 50 cells in each colony.

### 3.7. Wound Healing and Transwell Migration Assay

Cell migration ability was examined using wound healing and the transwell migration assay. For the wound healing assay, Hela cells were allowed to grow to 80–90% confluence in 6-well plates. A wound was built afterwards across the cell monolayer using a 10 μL pipette tip. After rinsing with PBS, the cells were incubated for another 24 h using serum-free medium. The images of wound closures were captured (0 and 24 h) using a fluorescence microscope (ZEISS, Oberkochen, Germany). For the transwell migration assay, Hela cells were seeded into transwell at a density of 2 × 10^4^ cells/well. After 24 h of incubation, Hela cells were treated with the tested sample for 24 h. The upper chamber was filled with 100 μL medium without FBS and the bottom chamber was filled with 500 μL medium with 20% FBS. After incubating for 24 h, the non-migrated Hela cells were cleared, and the invaded cells on the lower the membrane were stained with 1% crystal violet for 20 min. After that, the cell number was counted in five different visual fields under the fluorescence microscope.

### 3.8. Statistical Analysis

All data were represented as mean ± SD. The statistical differences were carried out using one-way analysis of ANOVA; *p* < 0.05 was considered to represent statistically significant differences.

## 4. Conclusions

In conclusion, one novel and nine known compounds were isolated and purified from the seeds of *O. violaceus*. Their structures were determined by the different spectroscopic methods. Among them, orychophragvioline A was confirmed as a new natural alkaloid with an unusual 1-methyl-4-phenyl-2,3-diketopiperazine skeleton connected with a guanidine group via an amide bond via single-crystal X-ray diffraction analysis. Furthermore, it was demonstrated to have excellent cytotoxicity against the Hela cell line. We also found that it could induce apoptosis and inhibit the proliferation, migration, and invasion of Hela cells significantly in a dose-dependent manner. Based on these results, orychophragvioline A could be regarded as a potential natural lead compound which is worthy of further structural reformation and modification to develop new analogs with a higher efficiency on the inhibition of cancer cells and a lower toxicity on normal cells. It is also expected to hold tremendous promise for fighting against cervical cancer.

## Figures and Tables

**Table 1:** ^1^H (600 MHz) and ^13^C (150 MHz) NMR data of compound **1** in DMSO-*d_6_*.

No.	*δ*_H_ (*J*, Hz)	*δ*_c_
2		156.5s
3		155.9s
5	3.67 m; 3.39 m	37.8t
6	3.67 m	54.8d
7	4.33 dd (12.9, 3.7); 3.67 m	48.1t
9		155.9s
11		156.5s
14	3.07 s	33.5q
1′		143.0s
2′, 6′	7.48 d (8.3)	124.8d
3′, 5′	7.89 d (8.3)	129.9d
4′		135.4s
7′		171.3s

**Table 2:** Cytotoxic activity of orychophragvioline A against four cancer cell lines in vitro ^1^.

Compounds	Cell Lines (IC_50_/μM)				
	Hela	U251	SW1990	4T1	L02
Orychophragvioline A	11.95 ± 1.52	18.54 ± 2.57	27.87 ± 1.25	23.54 ± 2.74	29.26 ± 3.12
Cisplatin	7.26 ± 0.25	10.26 ± 0.49	9.58 ± 0.84	8.29 ± 0.57	10.85 ± 0.98

^1^ The results were mean ± SD (*n* = 3).

## Data Availability

The data supporting the findings of this article are available from the corresponding author, Y.H., upon reasonable request.

## Supplementary Materials

The following supporting information can be downloaded at: https://www.mdpi.com/article/10.3390/molecules30081759/s1. Figures S1–S8: Spectral data (IR, ^1^H and ^13^C NMR, COSY, HSQC, HMBC, HR-ESI-MS, and UV) of compound **1**. Table S1: X-ray crystallographic data for compound **1**. Figures S9–S26: Spectral data (^1^H and ^13^C NMR) of compound **2-10**. I (18) and new saponins 19–21.

## Abbreviations

The following abbreviations are used in this manuscript:

DMSO	Dimethyl sulfoxide
IR	Infrared spectrum
HR-ESI-MS	High-resolution electrospray ionization mass spectroscopy
NMR	Nuclear magnetic resonance
