# Supplementary material for: Orychophragvioline A, a Novel Alkaloid Isolated from Orychophragmus violaceus with Anti-Cervical Cancer Activity
Source: Molecules. 2025 Apr 14;30(8):1759. doi: 10.3390/molecules30081759 (PMC12029839; doi:10.3390/molecules30081759)
Supplement: Supplementary file 1 [file molecules-30-01759-s001.zip › molecules-3569637-supplementary.pdf]

Orychophragvioline A, a novel alkaloid isolated from Orychophragmus violaceus with anti-cervical cancer activity

Ya Li [1,†], Tonghe Liu [2,†], Guangjie Pan [3,†], Yihang Li [4], Guoxu Ma [2], Yong Hou [2,*], Nailiang Zhu [2,5,*], Xudong Xu [2,*]

[1] *Department of Gynecologic Cancer, Beijing Arion Cancer Center, Beijing 100070, China*

[2] *Key Laboratory of Bioactive Substances and Resource Utilization of Chinese Herbal Medicine, Ministry of Education; Institute of Medicinal Plant Development, Peking Union Medical College and Chinese Academy of Medical Sciences, Beijing 100193, China*

[3] *College of Chinese Medicine Material, Jilin Agricultural University, Changchun 130118, China*

[4] *Yunnan Key Laboratory of Southern Medicine Utilization, Yunnan Branch, Institute of Medicinal Plant Development, Peking Union Medical College and Chinese Academy of Medical Sciences, Jinghong 666100, China*

[5] *Department of Traditional Chinese Medicine Resources and Development, School of pharmacy, Xinyang Agricultural and Forestry University, Xinyang, 464000, China*

[1]Ya L., T. Liu and G. Pan contributed equally to this work.

*Correspongding authors.

**Correspongding authors.

***Correspongding authors.

E-mail address: houyongyzs@sina.com (Y. Hou);

2021260008@xyafu.edu.cn (N. Zhu);

xdxu@implad.ac.cn (X. Xu)

Content

Figure S1. IR spectrum of 1. 3

Figure S2. ^{1}H NMR (600 MHz) spectrum of 1 in DMSO-d_6. 3

Figure S3. ^{13}C NMR (150 MHz) spectrum of 1 in DMSO-d_6. 4

Figure S4. ^{1}H-^{1}H COSY (600 MHz) spectrum of 1 in DMSO-d_6. 4

Figure S5. HSQC (600 MHz) spectrum of 1 in DMSO-d_6. 5

Figure S6. HMBC (600 MHz) spectrum of 1 in DMSO-d_6. 5

Figure S7. HR-ESI-MS spectrum of 1. 6

Figure S8. UV spectrum of 1. 6

Table S1. X-ray crystallographic data for 1. 7

Figure S9. ^{1}H NMR (600 MHz) spectrum of 2 in DMSO-d_6. 8

Figure S10. ^{13}C NMR (150 MHz) spectrum of 2 in DMSO-d_6. 8

Figure S11. ^{1}H NMR (600 MHz) spectrum of 3 in DMSO-d_6. 9

Figure S12. ^{13}C NMR (150 MHz) spectrum of 3 in DMSO-d_6. 9

Figure S13. ^{1}H NMR (600 MHz) spectrum of 4 in DMSO-d_6. 10

Figure S14. ^{13}C NMR (150 MHz) spectrum of 4 in DMSO-d_6. 10

Figure S15. ^{1}H NMR (600 MHz) spectrum of 5 in DMSO-d_6. 11

Figure S16. ^{13}C NMR (150 MHz) spectrum of 5 in DMSO-d_6. 11

Figure S17. ^{1}H NMR (600 MHz) spectrum of 6 in DMSO-d_6. 12

Figure S18. ^{13}C NMR (150 MHz) spectrum of 6 in DMSO-d_6. 12

Figure S19. ^{1}H NMR (600 MHz) spectrum of 7 in DMSO-d_6. 13

Figure S20. ^{13}C NMR (150 MHz) spectrum of 7 in DMSO-d_6. 13

Figure S21. ^{1}H NMR (600 MHz) spectrum of 8 in DMSO-d_6. 14

Figure S22. ^{13}C NMR (150 MHz) spectrum of 8 in DMSO-d_6. 14

Figure S23. ^{1}H NMR (600 MHz) spectrum of 9 in DMSO-d_6. 15

Figure S24. ^{13}C NMR (150 MHz) spectrum of 9 in DMSO-d_6. 15

Figure S25. ^{1}H NMR (600 MHz) spectrum of 10 in DMSO-d_6. 16

Figure S26. ^{13}C NMR (150 MHz) spectrum of 10 in DMSO-d_6. 16

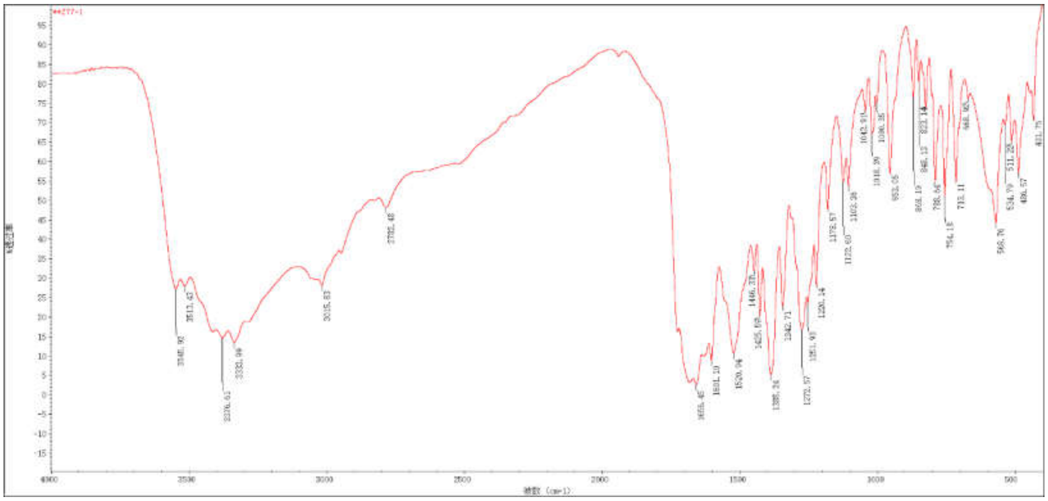

Figure S1. IR **spectrum of 1.**

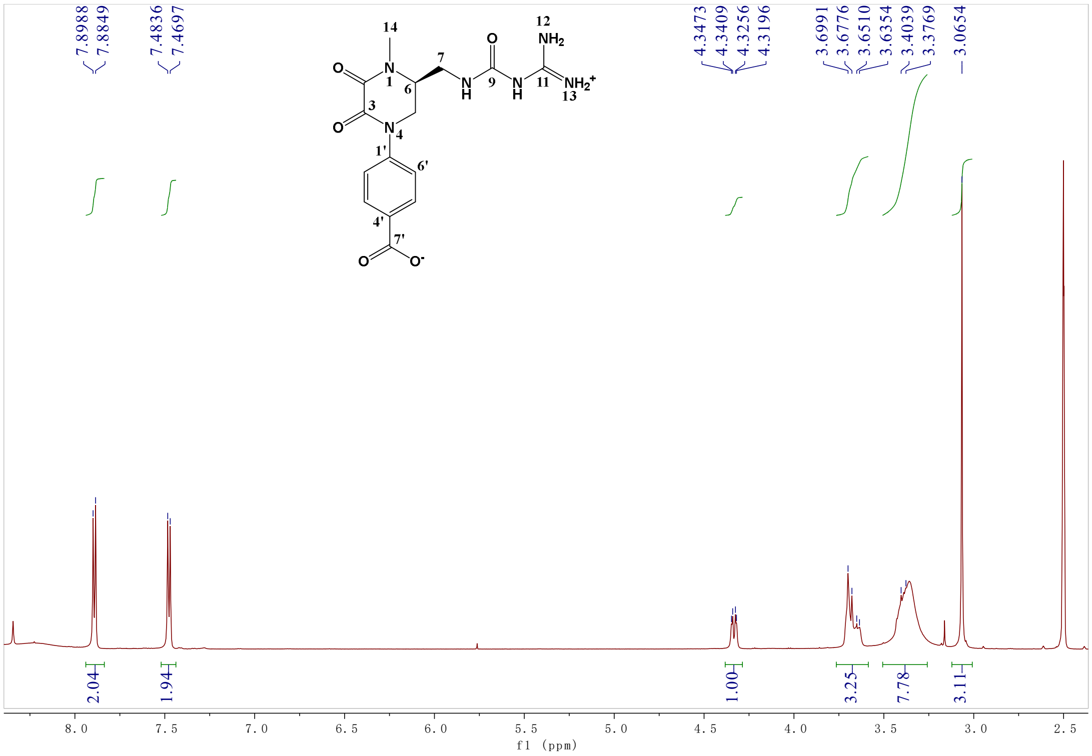

Figure S2. ^{1}H NMR (600 MHz) spectrum of 1 in DMSO-d_6.

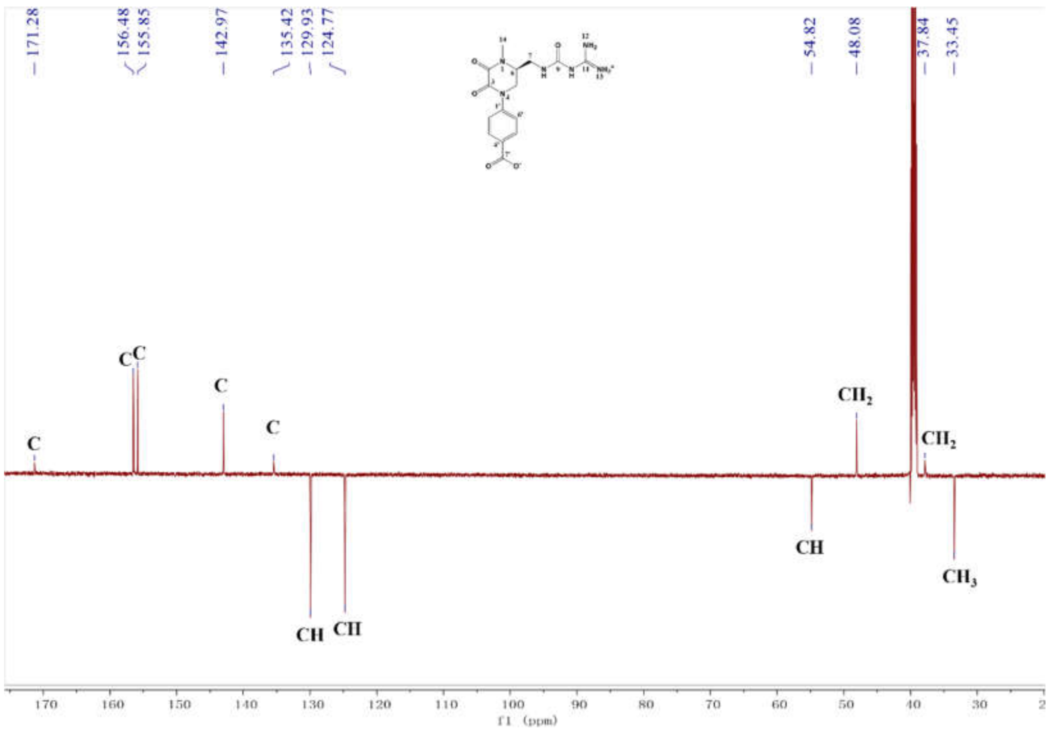

Figure S3. ^{13}C NMR (150 MHz) spectrum of 1 in DMSO-d_6.

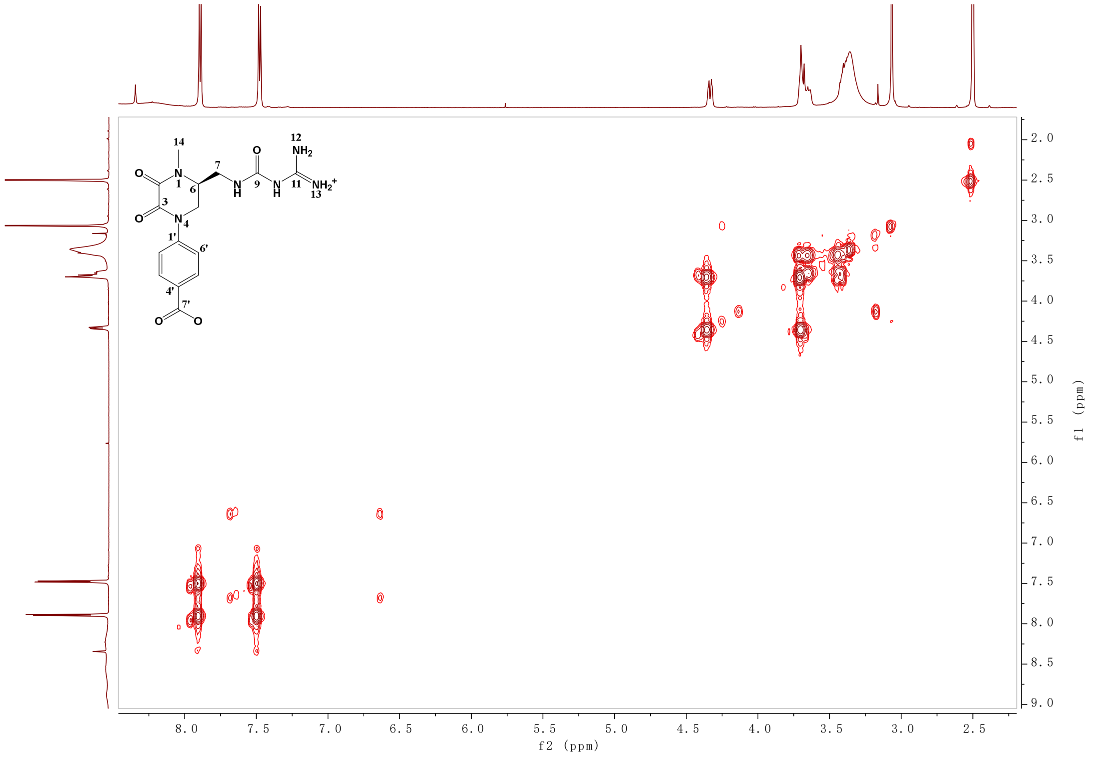

Figure S4. ^{1}H-^{1}H COSY (600 MHz) spectrum of 1 in DMSO-d_6.

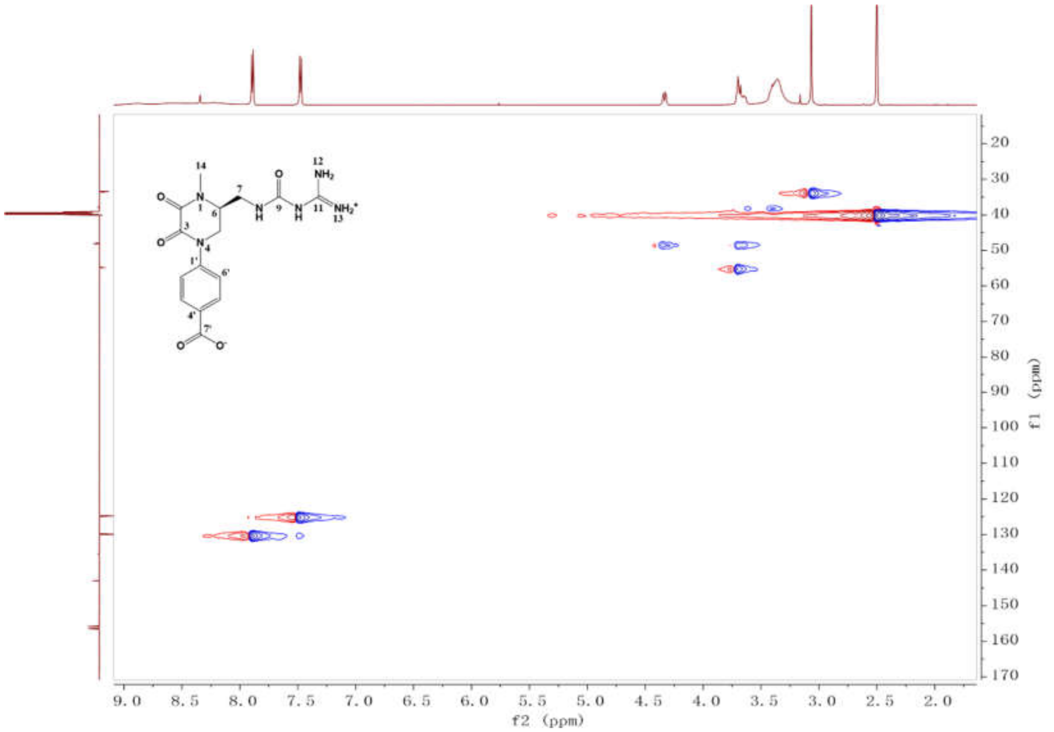

Figure S5. HSQC (600 MHz) spectrum of 1 in DMSO-d_6.

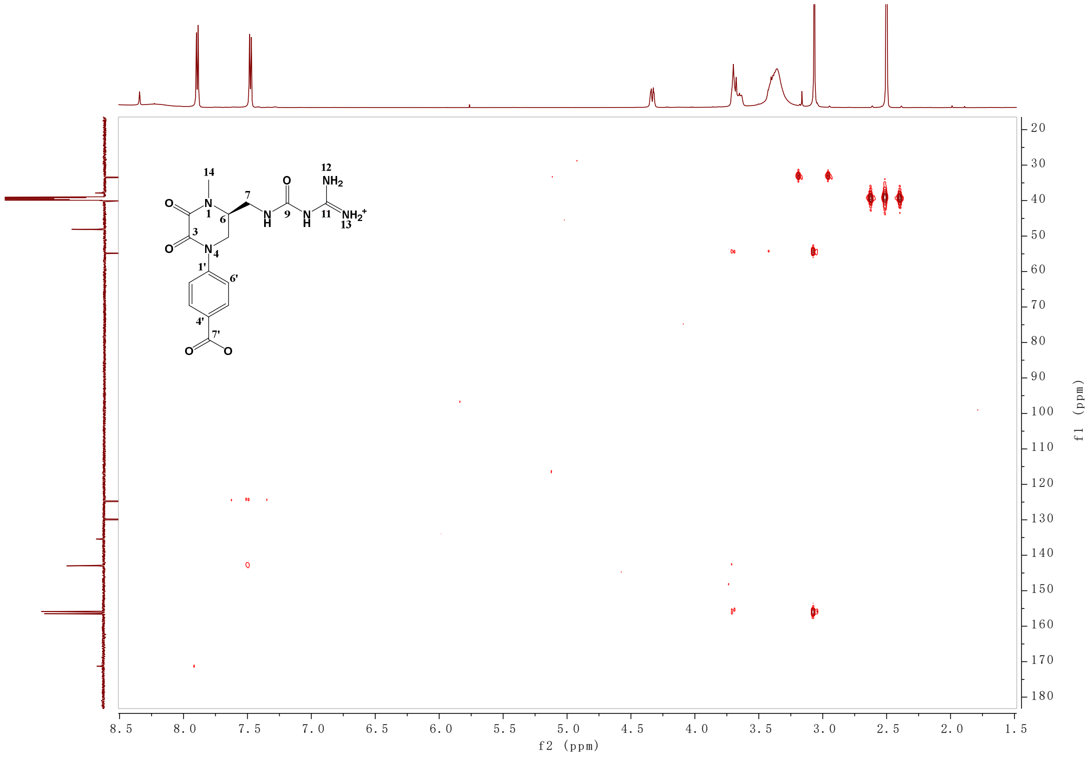

Figure S6. HMBC (600 MHz) spectrum of 1 in DMSO-d_6.

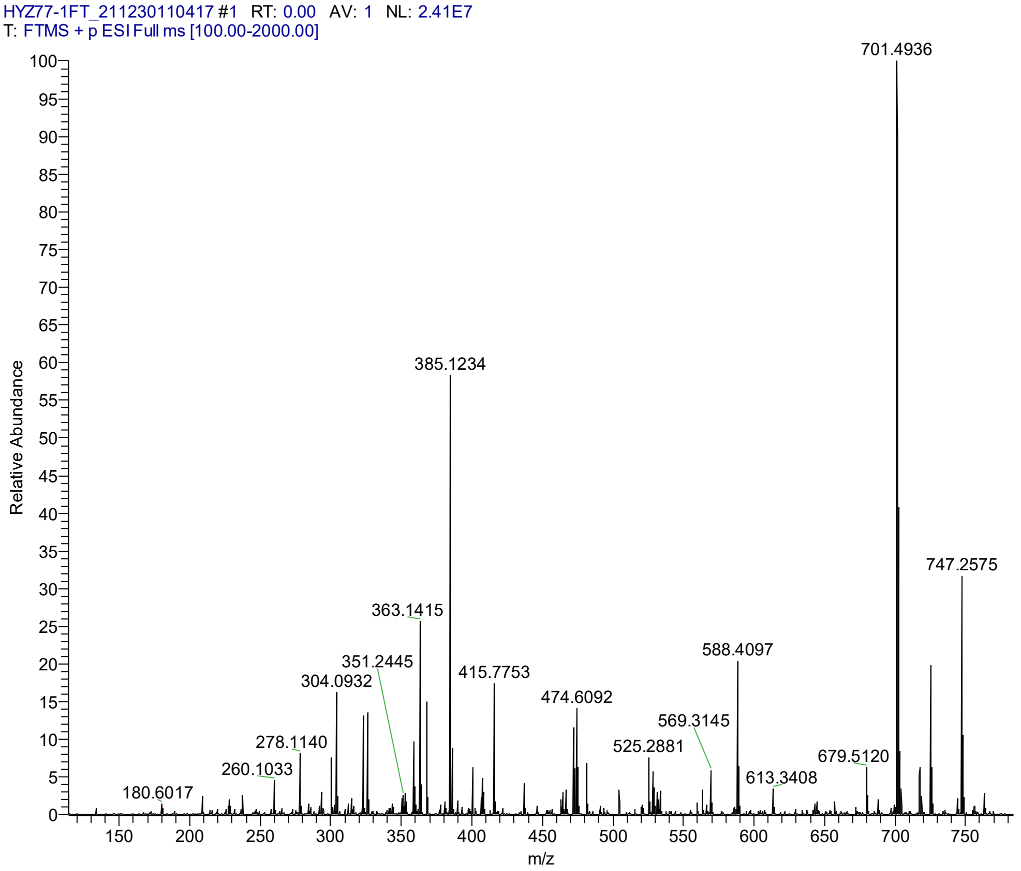

Figure S7. HR-ESI-MS spectrum of 1.

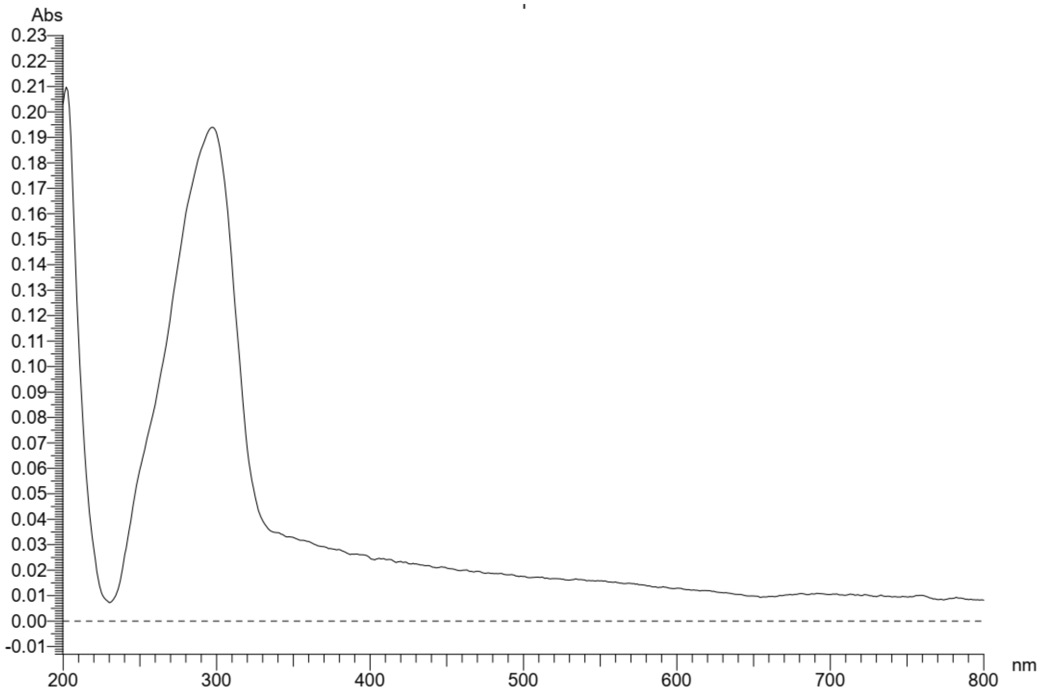

Figure S8. UV spectrum of 1.

Table S1. X-ray crystallographic data for 1.

Formula weight	398.38
Temperature/K	100.01(10)
Crystal system	triclinic
Space group	P1
a/Å	7.55406(4)
b/Å	10.02375(6)
c/Å	12.59483(7)
α/°	109.8415(5)
β/°	90.2652(4)
γ/°	103.3279(5)
Volume/$Å^3$	869.319(9)
Z	2
ρ_{calc}g/cm^3	1.522
μ/mm^{-1}	1.042
F(000)	420.0
Crystal size/mm^3	0.22 × 0.17 × 0.15
Radiation	Cu Kα (λ = 1.54184)
2Θ range for data collection/°	7.492 to 153.62
Index ranges	$-9 \leq h \leq 9$, $-12 \leq k \leq 12$, $-14 \leq l \leq 15$
Reflections collected	37657
Independent reflections	6571 [R_{int} = 0.0198, R_{sigma} = 0.0111]
Data/restraints/parameters	6571/3/520
Goodness-of-fit on F^2	1.029
Final R indexes [I>=2σ (I)]	R_1 = 0.0254, wR_2 = 0.0706
Final R indexes [all data]	R_1 = 0.0255, wR_2 = 0.0706
Largest diff. peak/hole / e $Å^{-3}$	0.23/-0.26
Flack parameter	0.00(3)

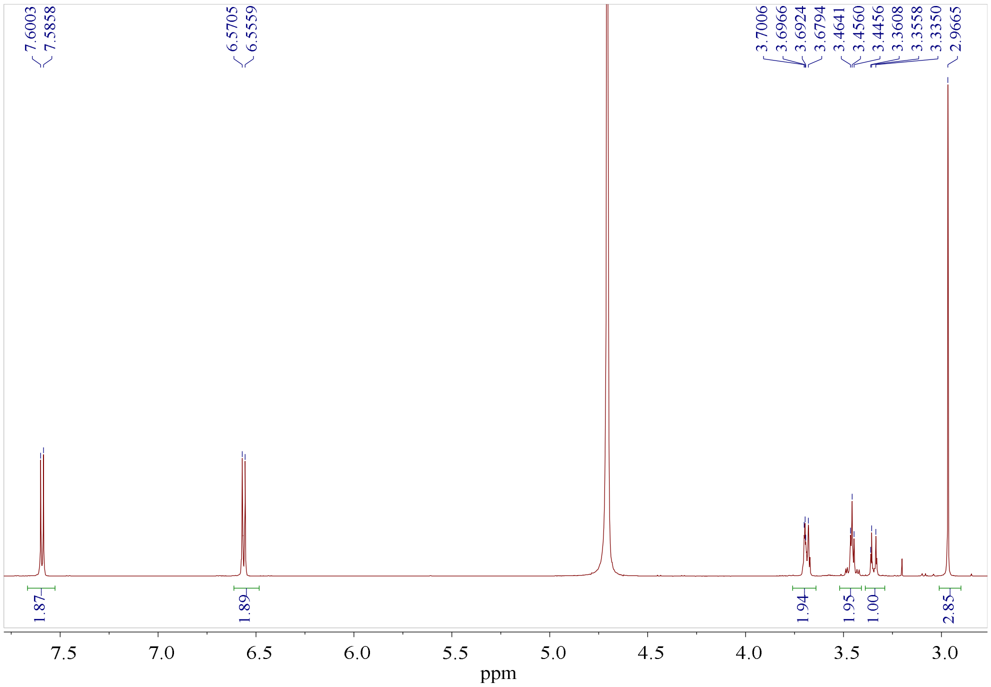

Figure S9. ^{1}H NMR (600 MHz) spectrum of 2 in DMSO-d_6.

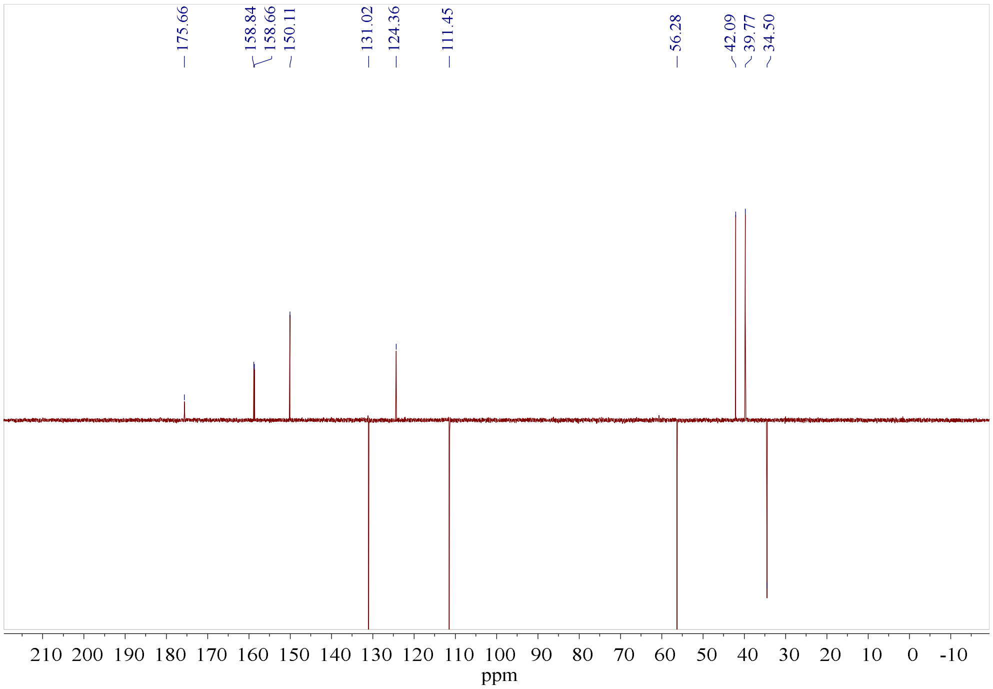

Figure S10. ^{13}C NMR (150 MHz) spectrum of 2 in DMSO-d_6.

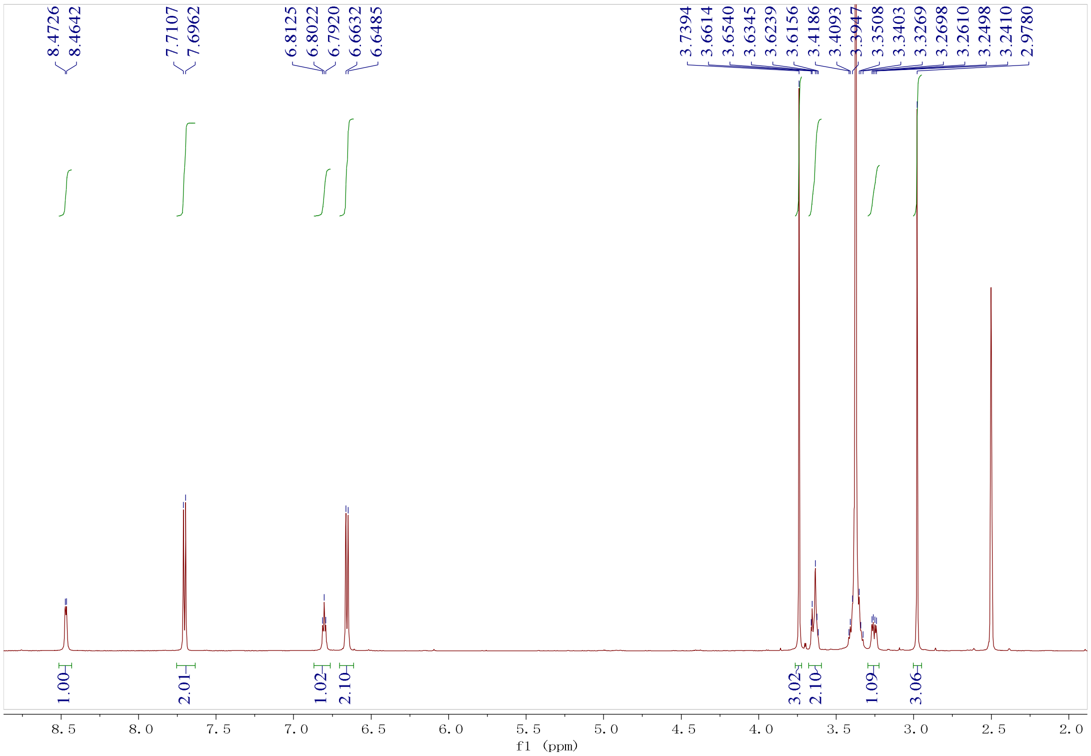

Figure S11. ^{1}H NMR (600 MHz) spectrum of 3 in DMSO-d_6.

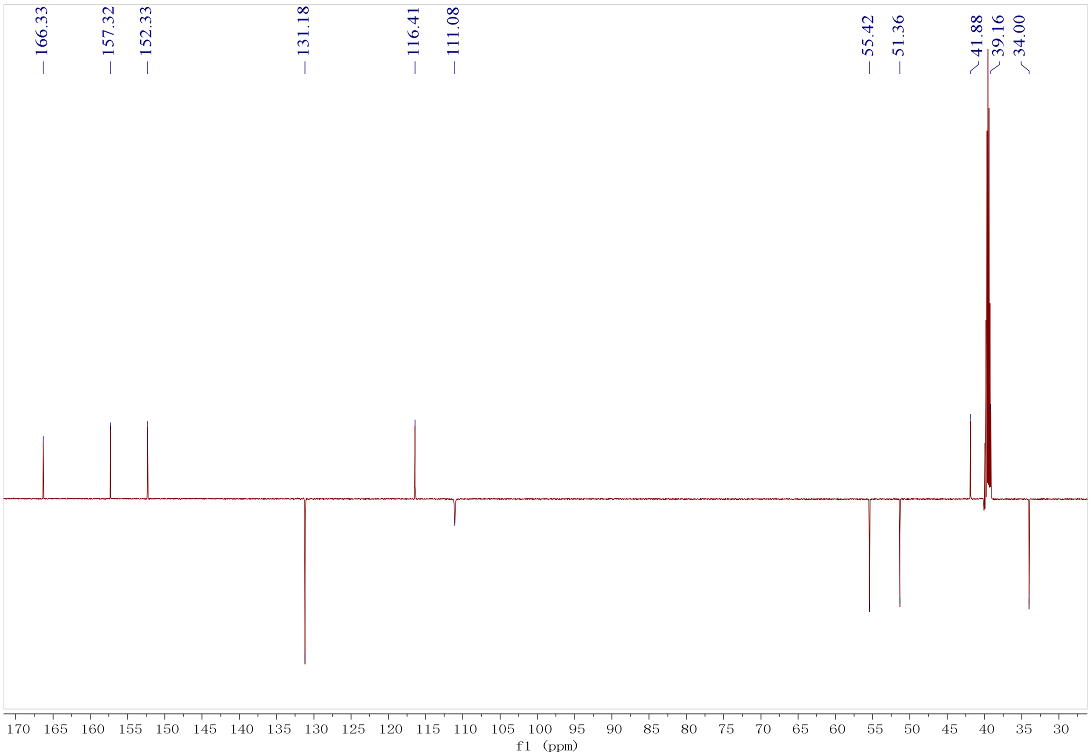

Figure S12. ^{13}C NMR (150 MHz) spectrum of 3 in DMSO-d_6.

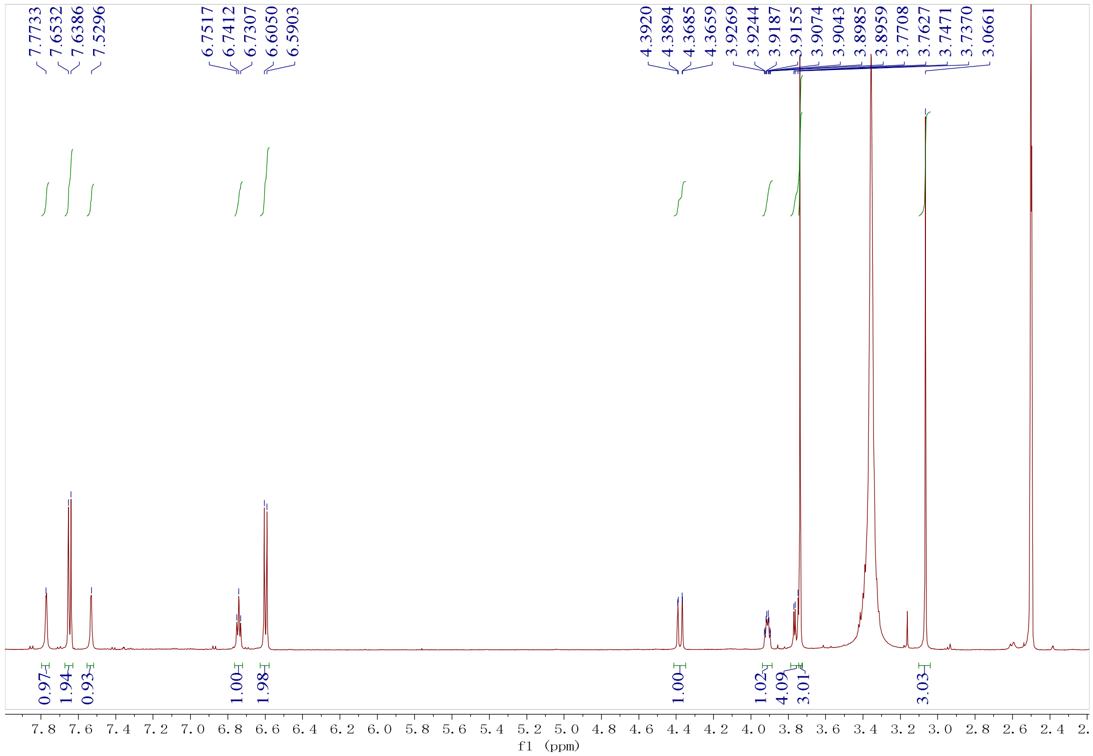

Figure S13. ^{1}H NMR (600 MHz) spectrum of 4 in DMSO-d_6.

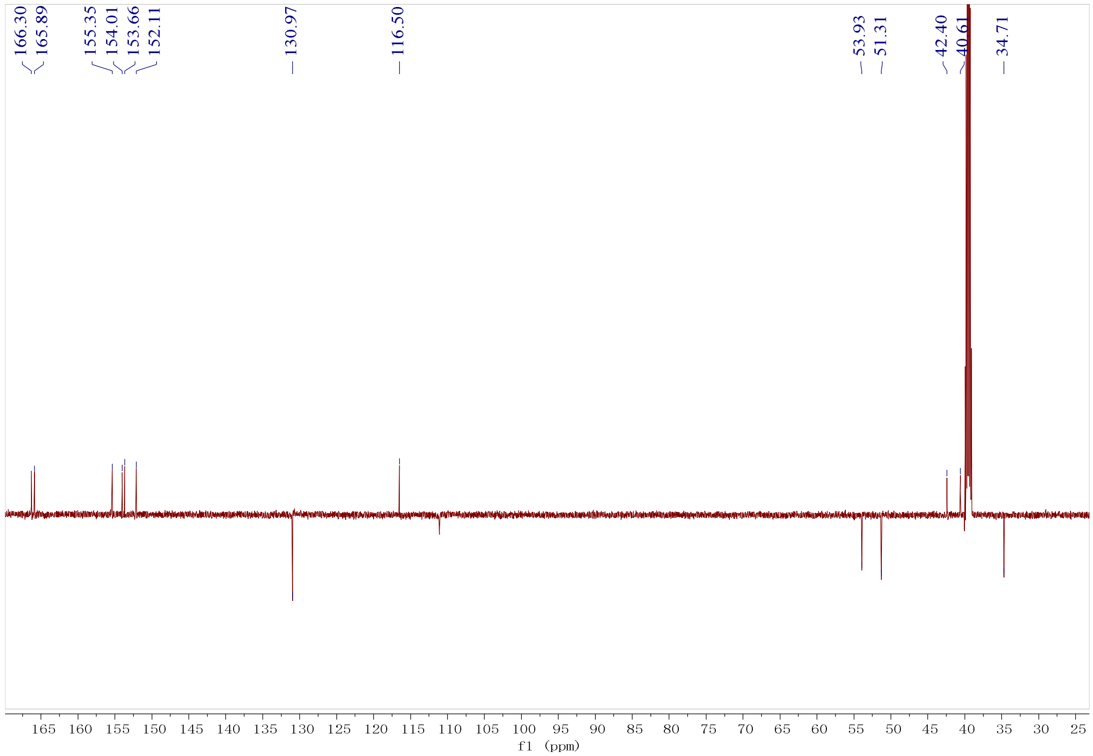

Figure S14. ^{13}C NMR (150 MHz) spectrum of 4 in DMSO-d_6.

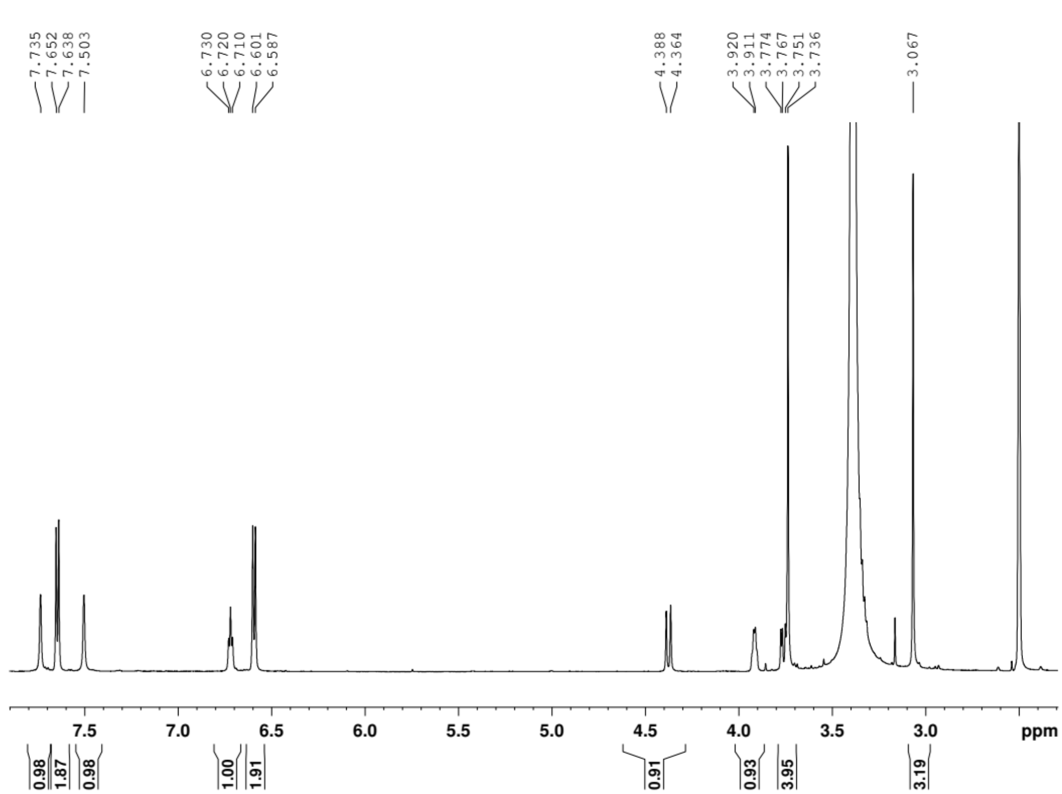

Figure S15. ^{1}H NMR (600 MHz) spectrum of 5 in DMSO-d_6.

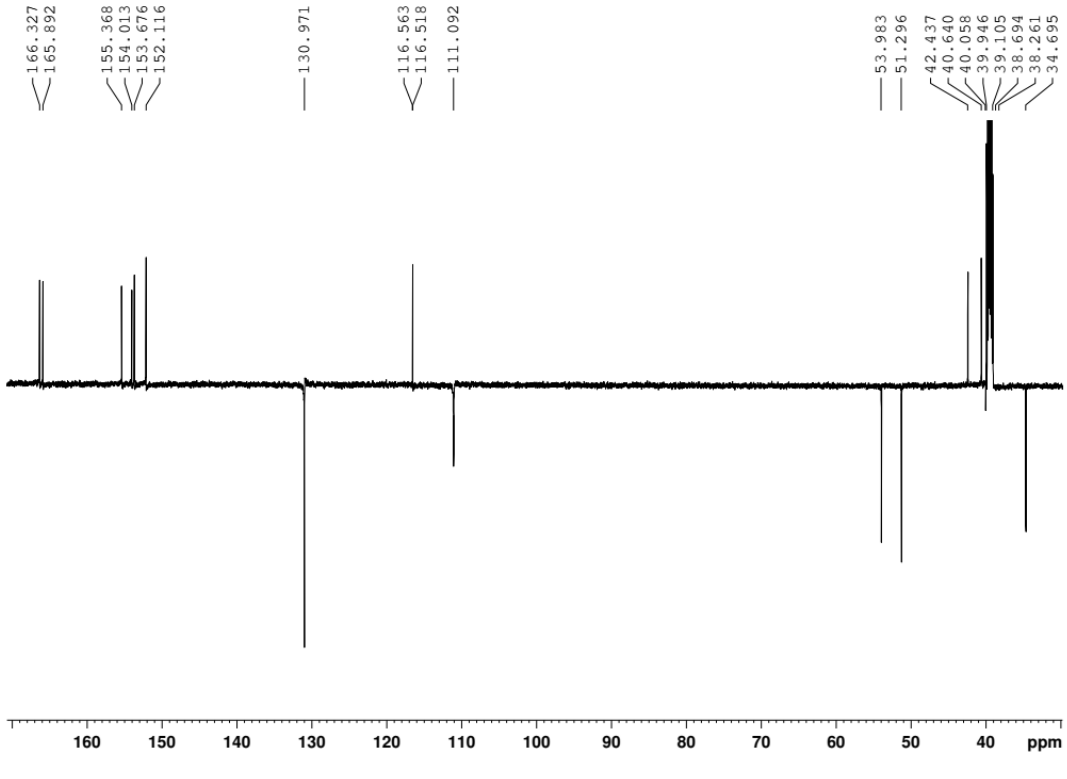

Figure S16. ^{13}C NMR (150 MHz) spectrum of 5 in DMSO-d_6.

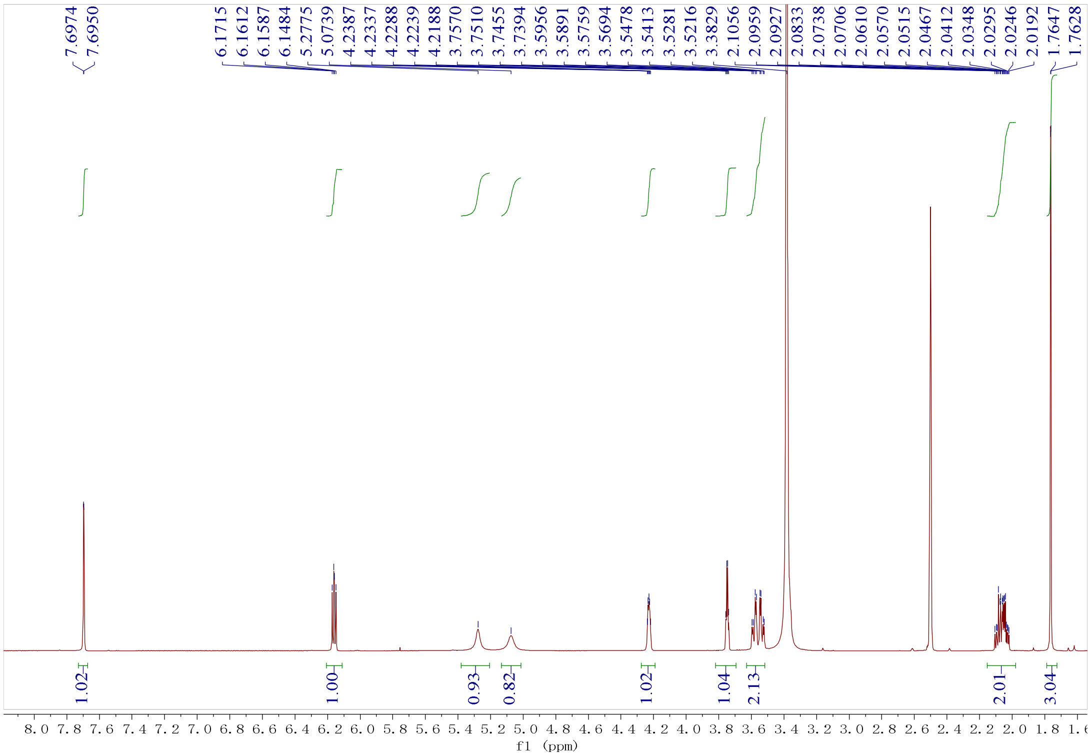

Figure S17. ^{1}H NMR (600 MHz) spectrum of 6 in DMSO-d_6.

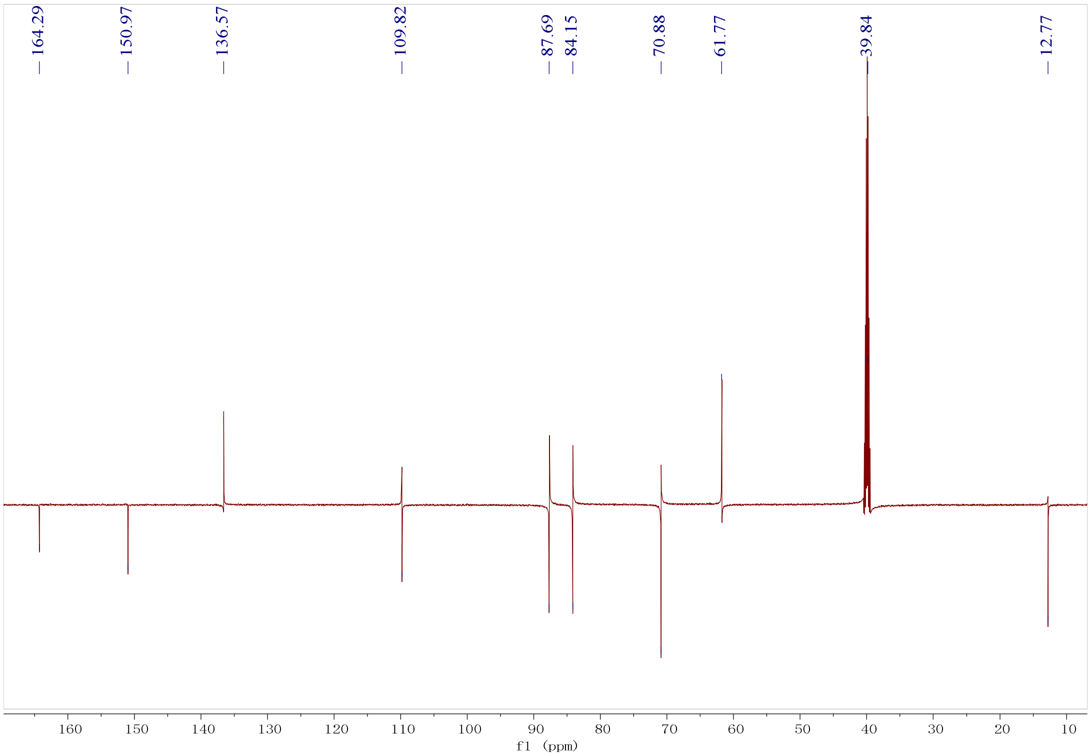

Figure S18. ^{13}C NMR (150 MHz) spectrum of 6 in DMSO-d_6.

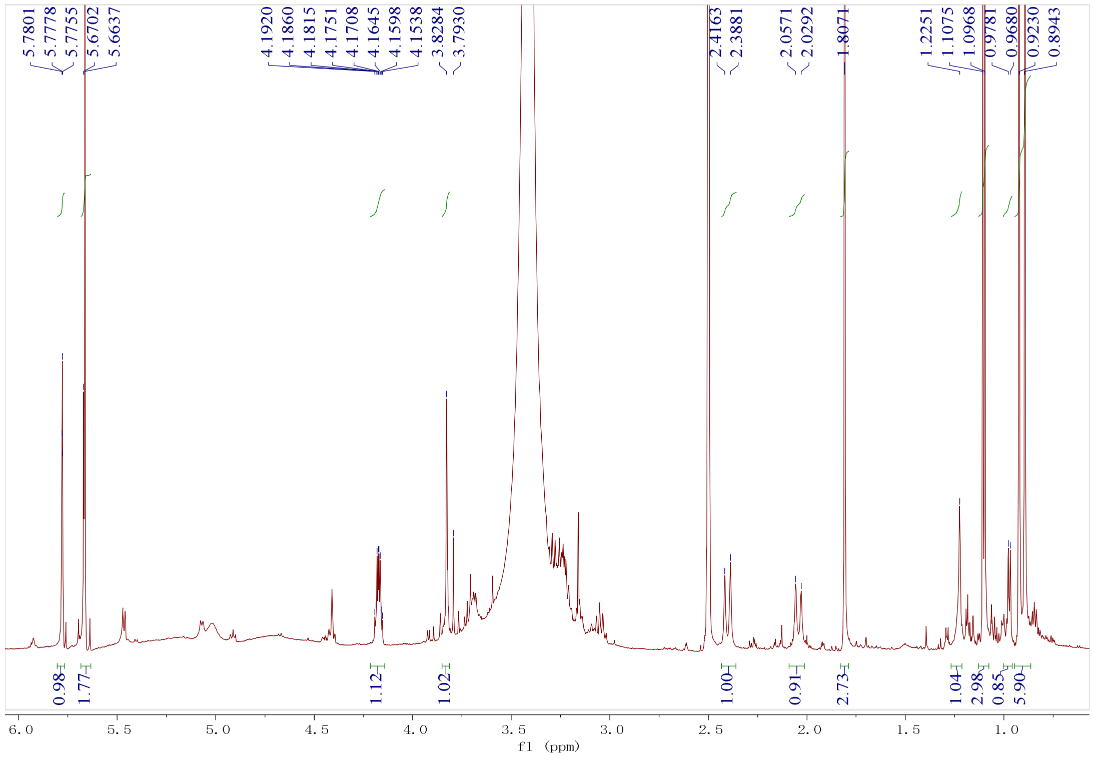

Figure S19. ^{1}H NMR (600 MHz) spectrum of 7 in DMSO-d_6.

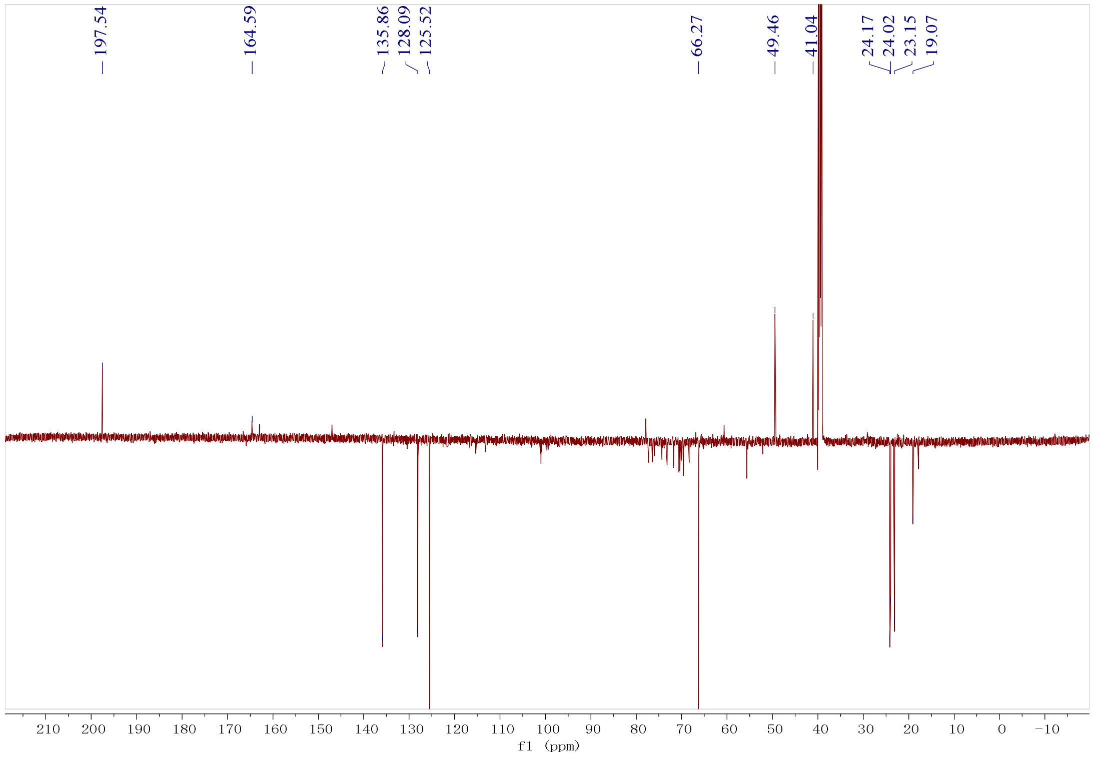

Figure S20. ^{13}C NMR (150 MHz) spectrum of 7 in DMSO-d_6.

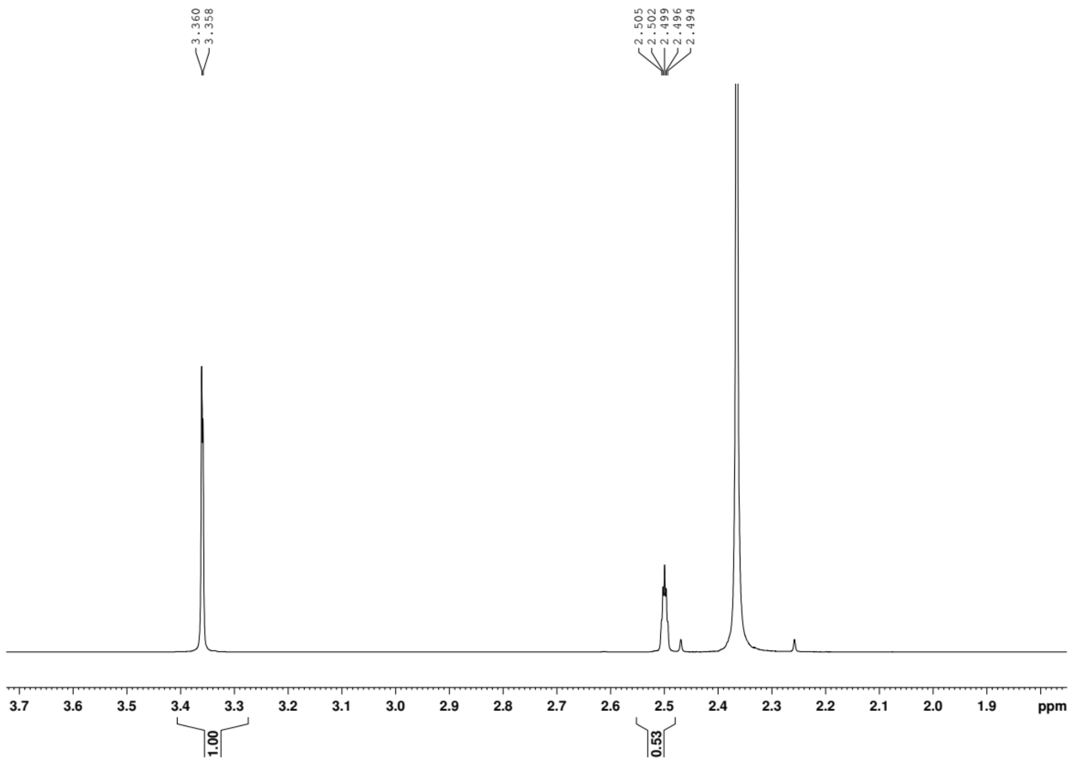

Figure S21. ^{1}H NMR (600 MHz) spectrum of 8 in DMSO-d_6.

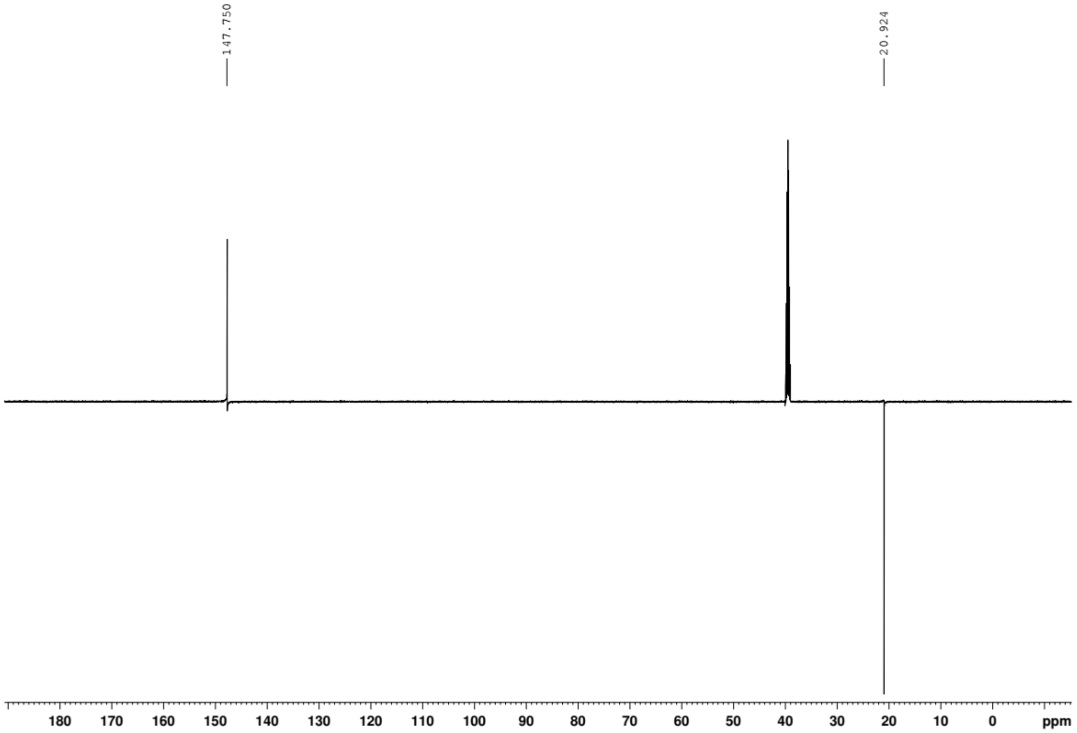

Figure S22. ^{13}C NMR (150 MHz) spectrum of 8 in DMSO-d_6.

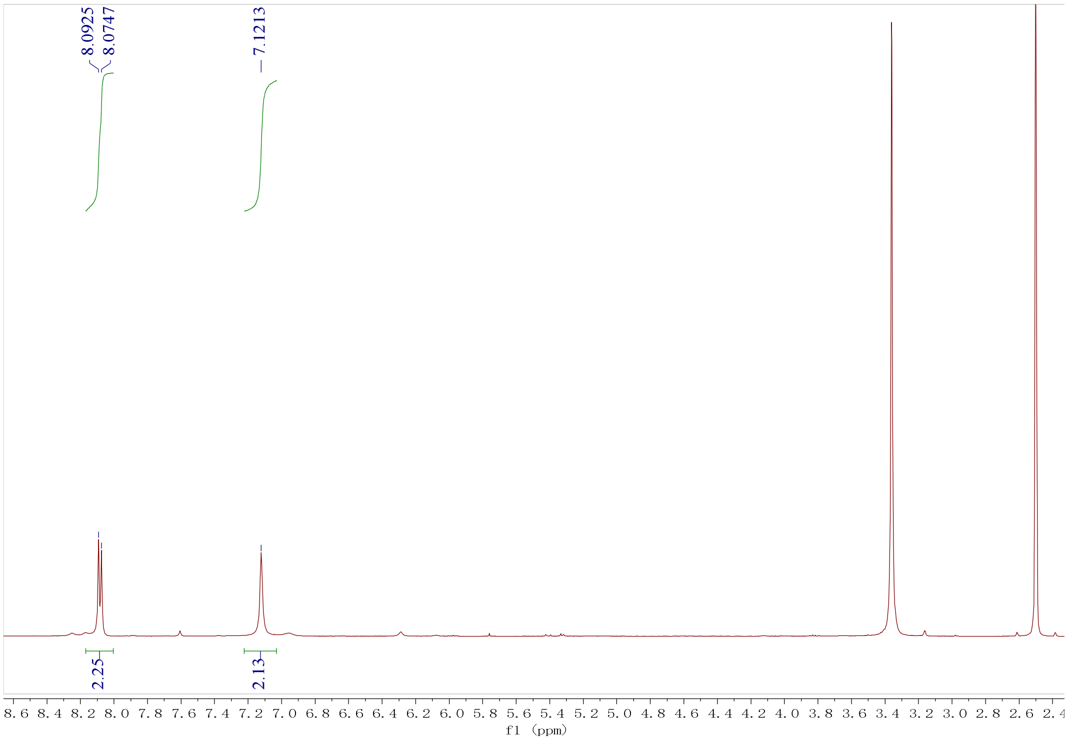

Figure S23. ^{1}H NMR (600 MHz) spectrum of 9 in DMSO-d_6.

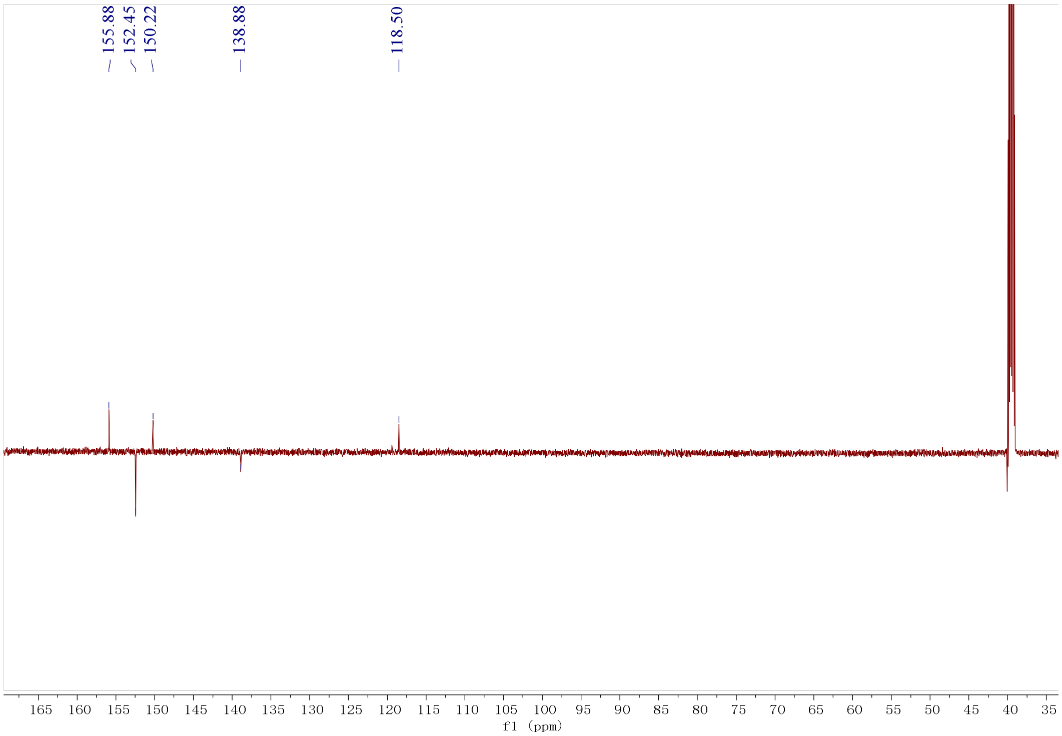

Figure S24. ^{13}C NMR (150 MHz) spectrum of 9 in DMSO-d_6.

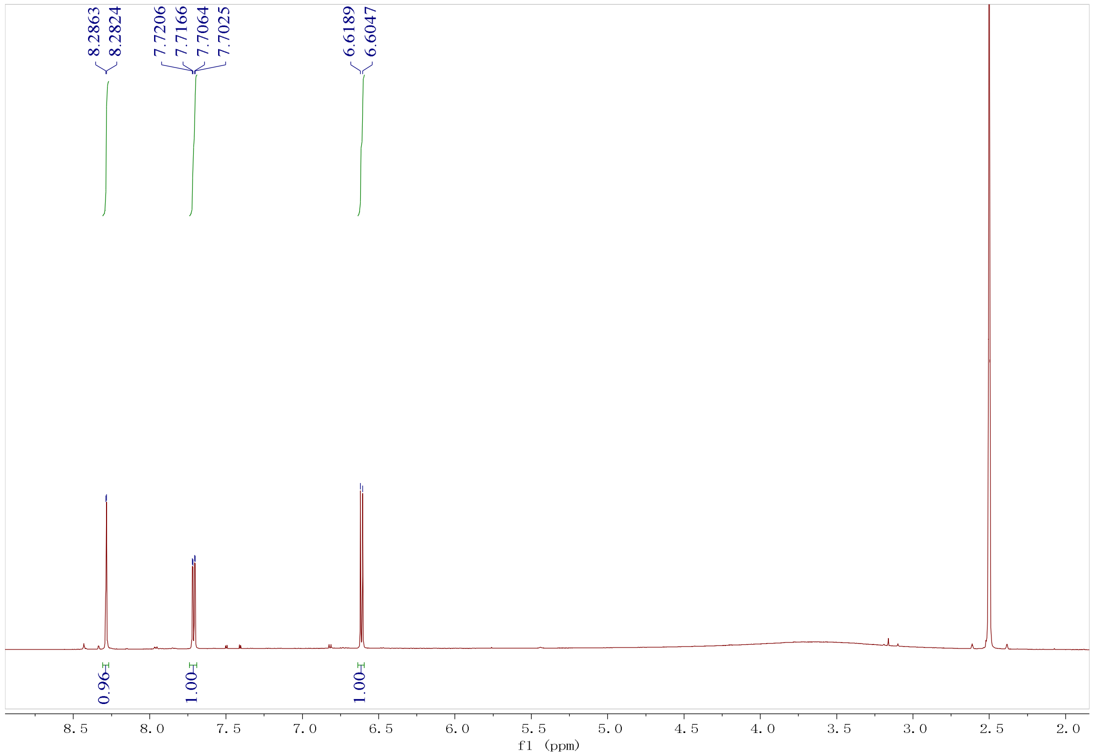

Figure S25. ^{1}H NMR (600 MHz) spectrum of 10 in DMSO-d_6.

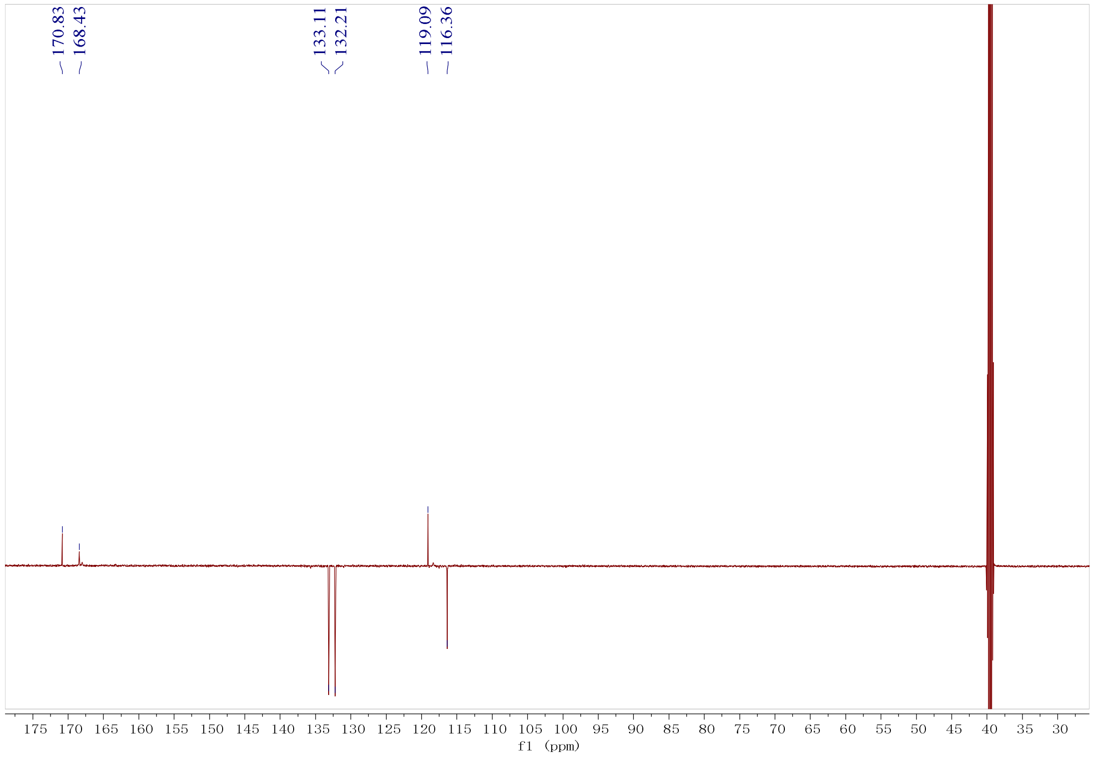

Figure S26. ^{13}C NMR (150 MHz) spectrum of 10 in DMSO-d_6.
